# Supplementary figures and images for: Use of a paediatric advice line for parents of infants recruited to a randomised controlled trial
Source: BMJ Paediatr Open. 2023 Feb 9;7(1):e001665. doi: 10.1136/bmjpo-2022-001665 (PMC9923309; doi:10.1136/bmjpo-2022-001665)

**Appendix A.** Participant recruitment timing based on date of birth in 3-monthly periods.

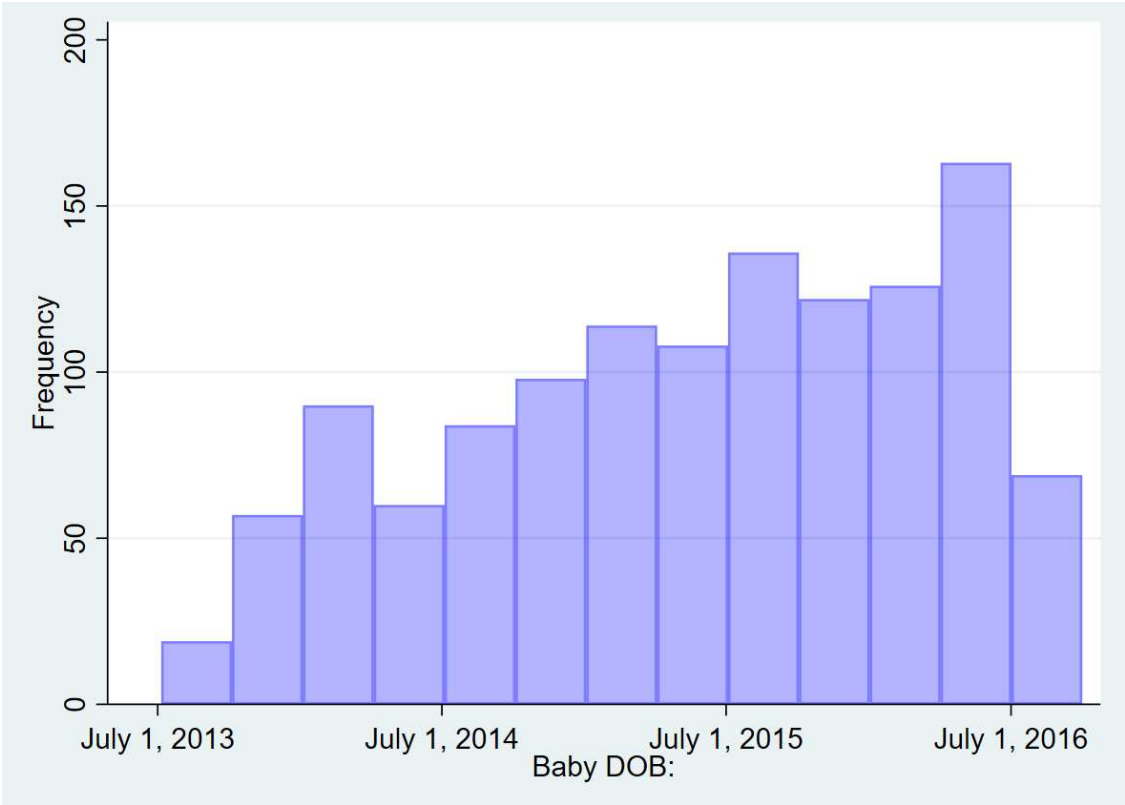

Supplement: Supplementary data [file bmjpo-2022-001665supp001.pdf]
