# Supplementary material for: Use of a paediatric advice line for parents of infants recruited to a randomised controlled trial
Source: BMJ Paediatr Open. 2023 Feb 9;7(1):e001665. doi: 10.1136/bmjpo-2022-001665 (PMC9923309; doi:10.1136/bmjpo-2022-001665)

BMJ Paediatrics Open

BMJ Paediatrics Open is committed to open peer review. As part of this commitment we make the peer review history of every article we publish publicly available.

When an article is published we post the peer reviewers' comments and the authors' responses online. We also post the versions of the paper that were used during peer review. These are the versions that the peer review comments apply to.

The versions of the paper that follow are the versions that were submitted during the peer review process. They are not the versions of record or the final published versions. They should not be cited or distributed as the published version of this manuscript.

BMJ Paediatrics Open is an open access journal and the full, final, typeset and author-corrected version of record of the manuscript is available on our site with no access controls, subscription charges or pay-per-view fees (http://bmjpaedsopen.bmj.com).

If you have any questions on BMJ Paediatrics Open's open peer review process please email info.bmjpo@bmj.com

BMJ Paediatrics Open

Use of a paediatric advice line for parents of infants recruited to a randomised controlled trial

Journal:	*BMJ Paediatrics Open*
Manuscript ID	bmjpo-2022-001665
Article Type:	Original research letter
Date Submitted by the Author:	05-Sep-2022
Complete List of Authors:	Chan, Joyce; Murdoch Children's Research Institute, Infectious Diseases Research Group; The Royal Melbourne Hospital<br>Kirby, Jordan; Mercy Hospital for Women, Department of Obstetrics & Gynaecology<br>Gardiner, Kaya; Murdoch Children's Research Institute, Infectious Diseases Research Group<br>Abruzzo, Veronica; Murdoch Children's Research Institute, Infectious Diseases Research Group<br>Bannister, Samantha; Murdoch Children's Research Institute, Infectious Diseases Research Group; Royal Children's Hospital Research Institute, Infectious Diseases Unit<br>Bellamy, Karen; Australian Technical Advisory Group on Immunisation (ATAGI); Vaccine Safety Investigation Group (VSIG)<br>Brophy, Clare; Murdoch Children's Research Institute; The Peter Doherty Institute for Infection and Immunity<br>Clifford, Vanessa; Royal Children's Hospital Research Institute, Infectious Diseases Unit; Royal Children's Hospital Research Institute, Laboratory Services<br>Cox, Lianne; Murdoch Children's Research Institute; Royal Children's Hospital Research Institute,<br>Elborough, Hannah; Murdoch Children's Research Institute<br>Freyne, Bridget Freyne; University of Liverpool, Institute of Infection & Global Health<br>Summons, Freya; Murdoch Children's Research Institute, Infectious Diseases Research Group; The University of Melbourne, Department of Paediatrics<br>Morrison, Clare; Murdoch Children's Research Institute<br>Wall, Kate; Murdoch Children's Research Institute<br>Casalaz, Daniel; Mercy Hospital for Women, Department of Paediatrics<br>Curtis, Nigel; The University of Melbourne, Department of Paediatrics; Murdoch Childrens Research Institute, Infectious Diseases Research Group<br>Pittet, Laure F.; Murdoch Children's Research Institute, Infectious Diseases Research Group; The University of Melbourne, Department of Paediatrics
Keywords:	Technology, Health services research

SCHOLARONE™
Manuscripts

I, the Submitting Author has the right to grant and does grant on behalf of all authors of the Work (as defined in the below author licence), an exclusive licence and/or a non-exclusive licence for contributions from authors who are: i) UK Crown employees; ii) where BMJ has agreed a CC-BY licence shall apply, and/or iii) in accordance with the terms applicable for US Federal Government officers or employees acting as part of their official duties; on a worldwide, perpetual, irrevocable, royalty-free basis to BMJ Publishing Group Ltd ("BMJ") its licensees and where the relevant Journal is co-owned by BMJ to the co-owners of the Journal, to publish the Work in this journal and any other BMJ products and to exploit all rights, as set out in our licence.

The Submitting Author accepts and understands that any supply made under these terms is made by BMJ to the Submitting Author unless you are acting as an employee on behalf of your employer or a postgraduate student of an affiliated institution which is paying any applicable article publishing charge ("APC") for Open Access articles. Where the Submitting Author wishes to make the Work available on an Open Access basis (and intends to pay the relevant APC), the terms of reuse of such Open Access shall be governed by a Creative Commons licence – details of these licences and which Creative Commons licence will apply to this Work are set out in our licence referred to above.

Other than as permitted in any relevant BMJ Author's Self Archiving Policies, I confirm this Work has not been accepted for publication elsewhere, is not being considered for publication elsewhere and does not duplicate material already published. I confirm all authors consent to publication of this Work and authorise the granting of this licence.

Confidential: For Review Only

Supplementary Material:

Appendix A

Methods

Study setting

The MIS BAIR trial randomised healthy neonates to receive BCG-Denmark vaccine or no vaccine within 10 days of birth and were followed up regularly for up to five years with parent-completed questionnaires and blood sample collection. (1) A PAL was made available to the parents of all the infants recruited to MIS BAIR for the first two years of their participation as recognition for their contribution to the trial. Parents were given a mobile phone number and were told that it was intended only for non-urgent questions. They were told that the PAL operated during standard business hours on weekdays and that outside this time, a message could be left, and they would be contacted within one business day. A clinical member of the study team (doctor, nurse or midwife with paediatric experience) responded to all calls and provided advice (sometimes with emailed resources). The MIS BAIR PAL was staffed by a single nurse or midwife during business hours. Parents calling for a rash or skin condition were asked to send pictures by email or smartphone. If appropriate, follow-up calls were arranged with a nurse, a paediatrician, the participant's general practitioner, a specialist clinic or at the emergency department.

Data collection

Details of all calls were recorded by the clinical member answering the call using a Research Electronic Data Capture (REDCap) database, including principal concern(s) and outcome of the call. The data were subsequently cleaned, categorised and coded in a standardised manner by JC and JK.

Definitions

A PAL caller was defined as a parent who remained in the trial for at least 3 months (completed at least one of the 3-monthly questionnaires) and who called the PAL at least once during the first 2 years of their participation in the trial. A PAL non-caller was defined as a parent who remained in the trial for at least 3 months (completed at least one of the 3-

Confidential: For Review Only

monthly questionnaires) but who did not call the PAL. Parents who called exclusively with questions about the trial (including BCG vaccine injection site concerns) were defined as non-callers, and these calls were not included in the analysis. The principal concern was determined as the initial symptom discussed during the call, with other symptoms listed as additional concerns.

Patient and Public Involvement Statement

The MIS BAIR PAL was developed with the interests of both the public and the MIS BAIR participant families at the forefront of the study design. The primary outcome of the study was to observe parental use and experience of a provided medical advice telephone line with the aim to improve patient and parental trial engagement. Whilst parents were not directly involved with the study design, the MIS BAIR PAL was developed with aims of empowering parents and guiding informed consent. Parental satisfaction was not recorded and will form part of future study improvements.

Statistical analysis

The sociodemographic characteristics of PAL callers were compared with non-callers using chi-square tests, Fisher's exact tests, independent samples t-tests and Wilcoxon-Mann-Whitney tests. Univariate logistic regression was used to identify independent factors associated with the use of the PAL. All significant predictors ($p < 0.20$) were then included in a stepwise backward multivariate analysis. Statistical analysis was done using Stata version 16.

Confidential: For Review Only

Appendix B

Consort diagram for the MIS BAIR PAL participants.

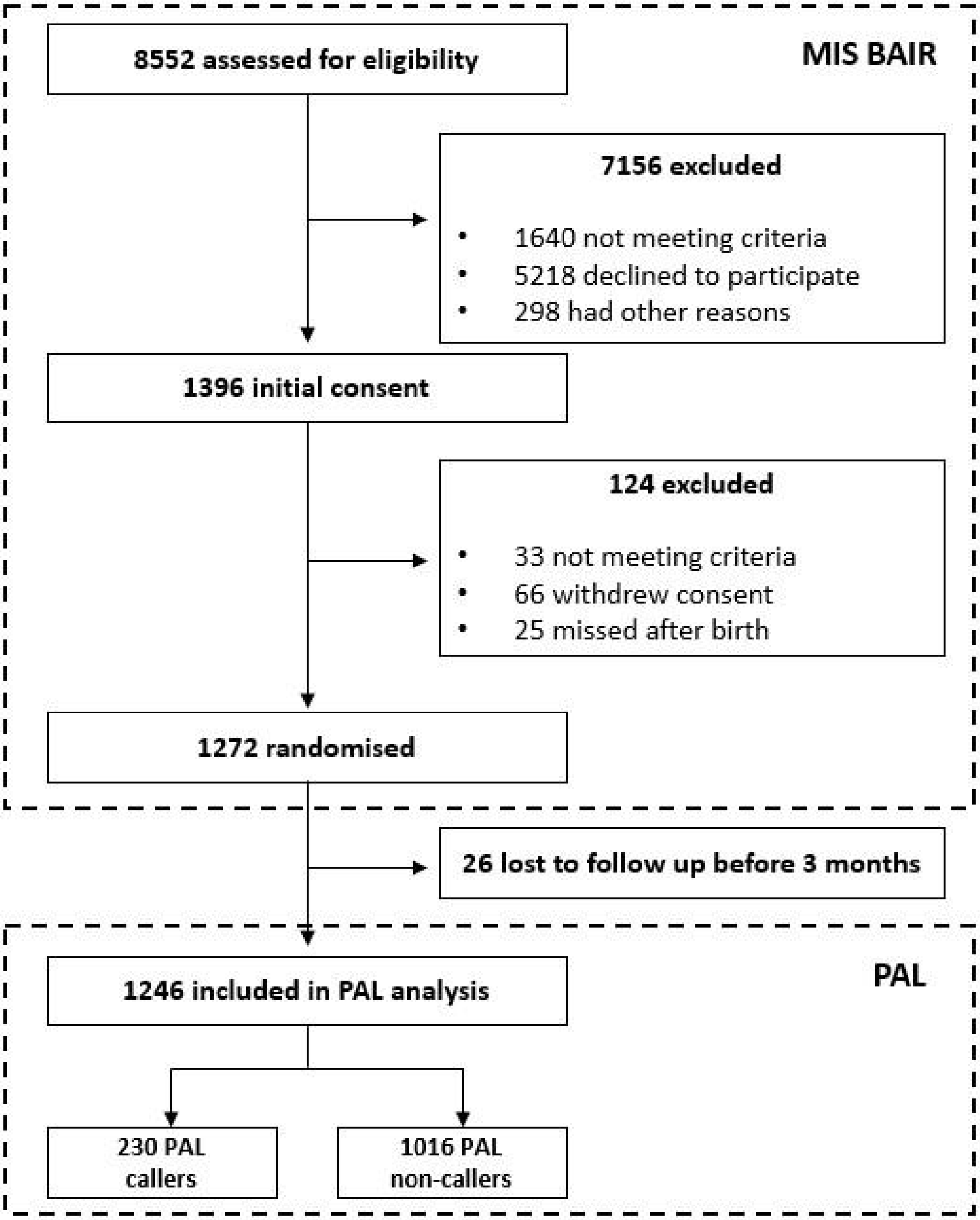

Appendix C

Supplementary Table 1. MIS BAIR PAL study parent and infant demographics, categorised as PAL callers and PAL non-callers.

	All participants (n=1246)	PAL callers (n=230)	PAL non-callers (n=1016)	P-value
**Sex of child**				
**Male**	628 (50.4%)	117 (50.9%)	511 (50.3%)	0.9
**Female**	618 (49.6%)	113 (49.1%)	505 (49.7%)	
**First time parent**				
**Yes**	649 (52.1%)	140 (60.9%)	509 (50.1%)	0.003
**No**	597 (47.9%)	90 (39.1%)	507 (49.9%	
**Number of siblings**				
**0**	649 (52.1%)	140 (60.9%)	509 (50.1%)	0.02
**1**	388 (31.1%)	69 (30.0%)	319 (31.4%)	
**2**	150 (12.0%)	15 (6.5%)	135 (13.3%)	
**3**	34 (2.7%)	4 (1.7%)	30 (3.0%)	
**4**	13 (1.0%)	1 (0.4%)	12 (1.2%)	
**5**	10 (0.8%)	1 (0.4%)	9 (0.9%)	
**6**	0 (0.0%)	0 (0.0%)	0 (0.0%)	
**7**	2 (0.2%)	0 (0.0%)	2 (0.2%)	
**Birth method**				
**Vaginal**	800 (64.2%)	150 (65.2%)	650 (64.0%)	0.7
**Caesarean**	446 (35.8%)	80 (34.8%)	366 (36.0%)	
**Gestational age**				
**Weeks***	39.3 (38.4, 40.3)	39.4 (38.5, 40.5)	39.3 (38.4, 40.3)	0.1
**IVF or sperm/egg donor**				
**IVF/donor used**	20 (1.6%)	8 (3.5%)	12 (1.2%)	0.003
**No IVF/donor used**	333 (26.7%)	49 (21.3%)	284 (28.0%)	
**Unknown**	893 (71.7%)	173 (75.2%)	720 (70.9%)	
**Birthweight**				
**Birthweight (kg)***	3.4 (3.1, 3.7)	3.4 (3.1, 3.8)	3.4 (3.1, 3.7)	0.3
**Maternal age at delivery**				
**Years#**	32.7 (4.7)	33.3 (4.1)	32.6 (4.9)	0.06
**Mother's birthplace**				
**Australia**	902 (72.4%)	169 (73.5%)	733 (72.2%)	0.2
**Outside Australia**	344 (27.6%)	61 (26.5%)	283 (27.9%)	
**Maternal education**				
**No education/up to Year 10**	69 (5.5%)	5 (2.2%)	64 (6.3%)	<0.001

Confidential: For Review Only

**Year 12/trade**	326 (26.2%)	39 (17.0%)	287 (28.1%)	
**University**	848 (68.1%)	185 (80.4%)	663 (65.3%)	
**Unknown**	3 (0.2%)	1 (0.4%)	2 (0.2%)	
**Breastfed**				
**Breastfed**	927 (74.4%)	187 (81.3%)	740 (72.8%)	0.03
**Not breastfed**	37 (3.0%)	2 (0.9%)	35 (3.4%)	
**Unknown**	282 (22.6%)	41 (17.8%)	241 (23.7%)	
**Received BCG**				
**Yes**	622 (49.9%)	119 (51.7%)	503 (49.5%)	0.4
**No**	624 (50.1%)	111 (48.3%)	513 (50.5%)	

Reported as number (%) and p-value calculated using chi-square or Fisher's exact test, unless otherwise noted.

* Reported as median (IQR). P-value calculated using Wilcoxon-Mann-Whitney test.

# Reported as mean (SD). P-value calculated using independent samples t-test.

Confidential: For Review Only

Title: Use of a paediatric advice line for parents of infants recruited to a randomised controlled trial

Corresponding Author: Joyce Chan, Murdoch Children's Research Institute, 50 Flemington Rd, Parkville VIC 3052, Australia. Email: joyce.chan@mcri.com.au Telephone: (+61 33) 8341 6200

Authors:

Joyce Chan [1, 2], Jordan Kirby [3] (joint first authors), Kaya Gardiner [1], Veronica Abruzzo [1], Samantha Bannister [1, 4], Karen Bellamy [5, 6], Clare Brophy [7, 8], Vanessa Clifford [4, 9], Lianne Cox [7], Hannah Elborough [7], Bridget Freyne [10], Freya Summons [1, 11], Clare Morrison [7], Kate Wall[7], Dan Casalaz [3], Nigel Curtis [1, 11], Laure F Pittet [1, 11] (joint last authors) on behalf of the MIS BAIR trial group

[1] Infectious Diseases Research Group, Murdoch Children's Research Institute, Parkville, Victoria, Australia

[2] The Royal Melbourne Hospital, Parkville, Victoria, Australia

[3] Mercy Hospital for Women, Heidelberg, Victoria, Australia

[4] Infectious Diseases Unit, The Royal Children's Hospital, Parkville, Victoria, Australia

[5] Australian Technical Advisory Group on Immunisation (ATAGI), Australia

[6] Vaccine Safety Investigation Group (VSIG), Therapeutic Goods Administration (TGA), Australia

[7] Murdoch Children's Research Institute, Parkville, Victoria, Australia

[8] Department of Medicine, The Doherty Institute, The University of Melbourne, Melbourne, Victoria, Australia

[9] Laboratory Services, The Royal Children's Hospital, Parkville, Victoria, Australia

[10] Institute of Infection & Global Health, University of Liverpool, United Kingdom

[11] Department of Paediatrics, The University of Melbourne, Parkville, Victoria, Australia

Keywords: Infectious Disease Medicine, Paediatrics, Technology, Child Health, Health services research

Confidential: For Review Only

Funding:

The MIS BAIR trial is funded by research grants from the National Health and Medical Research Council of Australia (GNT1051228 and GNT1099676), The University of Melbourne, The Royal Children's Hospital Foundation, the Murdoch Children's Research Institute, Mercy Health, the Myers Foundation, the European Society of Pediatric Infectious Diseases, the Nossal Institute for Global Health, and the Aranday Foundation. Dr Laure Pittet is supported by the Swiss National Science Foundation (Early Postdoc Mobility Grant number P2GEP3_178155).

Contribution Statement:

JC and JK contributed equally to this paper and are joint first authors. JC and JK transcribed the call data collected, performed statistical analysis and wrote the letter. NC and LP contributed equally to this paper and are joint last authors. NC and LP were integral to the study funding, design and letter production. All other authors were integral in data collection and management of the paediatric advice line, including taking calls and providing advice where necessary. All authors reviewed and approved the final letter.

Acknowledgments:

The authors thank the MIS BAIR participants and their families for their involvement in the trial. We also thank our recruitment and follow-up teams.

Conflict of Interest:

There are no reportable conflicts of interest.

Ethics Approval:

The PAL was included in the MIS BAIR ethical and governance approval from Mercy Health Human Research Ethics Committee (HREC, No. R12-28) and Royal Children's Hospital HREC (No. 33025) with additional governance approval from Barwon Health and St John of God, Geelong, Victoria. Signed informed consent was obtained from the parents of all participants.

Twitter:

Confidential: For Review Only

Jordan Kirby @JJKirbyMD, Nigel Curtis @nigeltwitt, Laure Pittet @PittetLaure

Words: 518

For the benefit of parents of participants in the Melbourne Infant Study: BCG for Allergy and Infection Reduction (MIS BAIR) randomised controlled trial (RCT) (1), we established a paediatric advice line (PAL), providing easy access to non-urgent, telephone-based paediatric medical advice. (2) The MIS BAIR PAL is among the first to be associated with a large RCT. Here we report the use of this PAL, including the number and types of medical concerns addressed, and compare the sociodemographic characteristics of those parents who used the PAL to those who did not.

In the MIS BAIR trial, infants were randomised to receive either the Bacillus Calmette-Guérin (BCG)-Denmark vaccine within 10 days of birth or no BCG vaccine to determine the off-target effects of this vaccine on allergies, eczema, infections and asthma. Participants were followed up regularly for up to five years. Here we report the use of the PAL by the parents of 1246 participants in the first two year of the trial (consort diagram, methodology and demographics detailed in supplementary appendices A and B).

The PAL was used by 230 (18.5%) of participants, with a total of 586 calls during the 2-year study period. The reasons for calling the PAL were: dermatological (24%); gastrointestinal (18%); disturbances in feeding, sleeping and crying (14%); respiratory (7%); and developmental/neurological (6%) with varied age-related distributions (Figure 1).

The sociodemographic characteristics of PAL callers and non-callers were compared (Supplementary Table 1, appendix C). Sociodemographic predictors via univariate and multivariate analyses are outlined in Table 1. Univariate analysis revealed that first-time parents were more likely to call than non-first-time parents (140/649 (21.6%) vs. 90/597 (15.1%), p=0.003), as were parents of infants who were breastfed (187/927 (20.2%) vs. 2/37 (5.4%), p=0.03) and mothers who were university-educated (185/848 (22%) vs. 44/395 (11%), p<0.001). PAL callers had a higher maternal age (mean 33.3 years, SD 4.1) compared with non-callers (mean 32.6 years, SD 4.9, p=0.06). In the final multivariate analysis, being a first-time parent remained a statistically significant predictor for calling the PAL (OR 1.4,

Confidential: For Review Only

95% CI 1.1 to 1.9), as did maternal university education (OR 3.3, 95% CI 1.3 to 8.4). Breastfeeding and higher maternal age were no longer predictive, likely due to them being confounded by higher maternal education status in the model. Parents of infants conceived through IVF or donor gametes were more likely to call (8/20 (40%) vs. 49/333 (14.7%), p=0.003). The infant's sex, delivery method, birthweight, maternal birthplace, or BCG vaccination status were not significantly different between callers and non-callers. This is consistent with the higher psychological stress and anxiety associated with first-time mothers of a higher maternal (3) and pregnancies following IVF and gamete donation. (4) Mothers with a university education might also feel more empowered to seek advice.

Confidential: For Review Only

Table 1. Univariate and multivariate analyses predicting PAL callers.

	Univariate model OR* (95% CI)	P-value	Multivariate model OR* (95% CI)	P-value
**Sex**				
**Male**	1.0 (0.8 – 1.4)	0.9		
**First time parent**				
**Yes**	1.5 (1.2 – 2.1)	0.003	1.4 (1.1 – 1.9)	0.02
**Number of siblings**				
**0**	Reference	Reference		
**1**	0.8 (0.6 – 1.1)	0.1		
**2**	0.4 (0.2 – 0.7)	0.002		
**3**	0.5 (0.2 – 1.4)	0.2		
**4**	0.3 (0.04 – 2.4)	0.3		
**5**	0.4 (0.05 – 3.2)	0.4		
**Birth method**				
**Vaginal**	1.1 (0.8 – 1.4)	0.7		
**Gestational age**				
**Weeks**	1.1 (1.0 – 1.2)	0.08		
**IVF or sperm/egg donor**				
**Yes**[#]	3.9 (1.5 – 9.9)	0.005		
**Birthweight**				
**<2500 g**	0.8 (0.3 – 1.8)	0.5		
**Birthweight (kg)**	1.2 (0.9 – 1.6)	0.2		
**Maternal age at delivery Years**	1.0 (1.0 – 1.1)	0.06		
**Mother's birthplace**				
**Australia**	1.1 (0.8 – 1.5)	0.7		
**Maternal education**				
**No education/up to Year 10**	Reference	Reference	Reference	
**Year 12/trade**	1.7 (0.7 – 4.6)	0.3	1.7 (0.6 – 4.5)	0.3
**University**	3.6 (1.4 – 9.0)	0.007	3.3 (1.3 – 8.4)	0.01
**Breastfed**				
**Yes**	4.4 (1.1 – 18.6)	0.04		
**Received BCG**				
**Yes**	1.1 (0.8 – 1.5)	0.5		

* Odds ratio calculated using logistic regression method. Univariate logistic regression was used to identify independent factors associated with the use of the PAL. All significant

Confidential: For Review Only

predictors from the univariate analysis ($p < 0.20$) were included in a stepwise backward multivariate analysis.

[#] Data missing for 893 participants. IVF was therefore not included in the multivariate analysis due to missing data resulting in loss of statistical power.

Overall, the MIS BAIR PAL demonstrates the demand for non-urgent paediatric medical advice within an RCT setting. The is the first Australian PAL to be combined with a multi-centre RCT, allowing an initial assessment of its use and the demographic data its consumers. Future studies should address whether advice lines incentivise trial participation and/or retention, include parental feedback and investigate further the influence of socioeconomic factors, such as maternal education, income and employment status.

Confidential: For Review Only

References:

1. Messina NL, Gardiner K, Donath S, Flanagan K, Ponsonby AL, Shann F et al. Study protocol for the Melbourne Infant Study: BCG for Allergy and Infection Reduction (MIS BAIR), a randomised controlled trial to determine the non-specific effects of neonatal BCG vaccination in a low-mortality setting. BMJ open. 2019 Dec 1;9(12):e032844.
2. Wang CJ, Ma J, Zuckerman B, Car J. The Opportunities for Telehealth in Pediatric Practice and Public Health. Pediatr Clin North Am. 2020 Aug;67(4):603-611. doi: 10.1016/j.pcl.2020.03.001
3. Aasheim V, Waldenström U, Hjelmstedt A, Rasmussen S, Pettersson H, Schytt E. Associations between advanced maternal age and psychological distress in primiparous women, from early pregnancy to 18 months postpartum. BJOG BJOG-INT J OBSTET GY. 2012 Aug;119(9):1108-16.
4. Hammarberg K, Fisher JR, Wynter KH. Psychological and social aspects of pregnancy, childbirth and early parenting after assisted conception: a systematic review. Hum. Reprod. 2008 Sep 1;14(5):395-414.

Confidential: For Review Only

BMJ Paediatrics Open

Use of a paediatric advice line for parents of infants recruited to a randomised controlled trial

Journal:	*BMJ Paediatrics Open*
Manuscript ID	bmjpo-2022-001665.R1
Article Type:	Original research
Date Submitted by the Author:	02-Oct-2022
Complete List of Authors:	Chan, Joyce; Murdoch Children's Research Institute, Infectious Diseases Research Group; The Royal Melbourne Hospital<br>Kirby, Jordan; Mercy Hospital for Women, Department of Obstetrics & Gynaecology<br>Gardiner, Kaya; Murdoch Children's Research Institute, Infectious Diseases Research Group<br>Abruzzo, Veronica; Murdoch Children's Research Institute, Infectious Diseases Research Group<br>Bannister, Samantha; Murdoch Children's Research Institute, Infectious Diseases Research Group; Royal Children's Hospital Research Institute, Infectious Diseases Unit<br>Bellamy, Karen; Australian Technical Advisory Group on Immunisation (ATAGI); Vaccine Safety Investigation Group (VSIG)<br>Brophy, Clare; Murdoch Children's Research Institute; The Peter Doherty Institute for Infection and Immunity<br>Clifford, Vanessa; Royal Children's Hospital Research Institute, Infectious Diseases Unit; Royal Children's Hospital Research Institute, Laboratory Services<br>Cox, Lianne; Murdoch Children's Research Institute; Royal Children's Hospital Research Institute,<br>Elborough, Hannah; Murdoch Children's Research Institute<br>Freyne, Bridget Freyne; University of Liverpool, Institute of Infection & Global Health<br>Summons, Freya; Murdoch Children's Research Institute, Infectious Diseases Research Group; The University of Melbourne, Department of Paediatrics<br>Morrison, Clare; Murdoch Children's Research Institute<br>Wall, Kate; Murdoch Children's Research Institute<br>Casalaz, Daniel; Mercy Hospital for Women, Department of Paediatrics<br>Curtis, Nigel; The University of Melbourne, Department of Paediatrics; Murdoch Childrens Research Institute, Infectious Diseases Research Group<br>Pittet, Laure F.; Murdoch Children's Research Institute, Infectious Diseases Research Group; The University of Melbourne, Department of Paediatrics
Keywords:	Technology, Health services research

SCHOLARONE™
Manuscripts

I, the Submitting Author has the right to grant and does grant on behalf of all authors of the Work (as defined in the below author licence), an exclusive licence and/or a non-exclusive licence for contributions from authors who are: i) UK Crown employees; ii) where BMJ has agreed a CC-BY licence shall apply, and/or iii) in accordance with the terms applicable for US Federal Government officers or employees acting as part of their official duties; on a worldwide, perpetual, irrevocable, royalty-free basis to BMJ Publishing Group Ltd ("BMJ") its licensees and where the relevant Journal is co-owned by BMJ to the co-owners of the Journal, to publish the Work in this journal and any other BMJ products and to exploit all rights, as set out in our licence.

The Submitting Author accepts and understands that any supply made under these terms is made by BMJ to the Submitting Author unless you are acting as an employee on behalf of your employer or a postgraduate student of an affiliated institution which is paying any applicable article publishing charge ("APC") for Open Access articles. Where the Submitting Author wishes to make the Work available on an Open Access basis (and intends to pay the relevant APC), the terms of reuse of such Open Access shall be governed by a Creative Commons licence – details of these licences and which Creative Commons licence will apply to this Work are set out in our licence referred to above.

Other than as permitted in any relevant BMJ Author's Self Archiving Policies, I confirm this Work has not been accepted for publication elsewhere, is not being considered for publication elsewhere and does not duplicate material already published. I confirm all authors consent to publication of this Work and authorise the granting of this licence.

Confidential: For Review Only

Use of a paediatric advice line for parents of infants recruited to a randomised controlled trial

Corresponding Author: Dr Joyce Chan (Murdoch's Children's Research Institute, 50 Flemington Rd, Parkville VIC 3052. Email: joyce.chan@mcri.com.au Telephone: (03) 8341 6200

Authors:

Joyce Chan [1, 2], Jordan Kirby [3] (joint first authors), Kaya Gardiner [1], Veronica Abruzzo [1], Samantha Bannister [1, 4], Karen Bellamy [5, 6], Clare Brophy [7, 8], Vanessa Clifford [4, 9], Lianne Cox [7], Hannah Elborough [7], Bridget Freyne [10], Freya Summons [1, 11], Clare Morrison [7], Kate Wall[7], Dan Casalaz [3], Nigel Curtis [1, 11], Laure F Pittet [1, 11] (joint last authors) on behalf of the MIS BAIR trial group

[1] Infectious Diseases Research Group, Murdoch Children's Research Institute, Parkville, Victoria, Australia

[2] The Royal Melbourne Hospital, Parkville, Victoria, Australia

[3] Mercy Hospital for Women, Heidelberg, Victoria, Australia

[4] Infectious Diseases Unit, The Royal Children's Hospital, Parkville, Victoria, Australia

[5] Australian Technical Advisory Group on Immunisation (ATAGI), Australia

[6] Vaccine Safety Investigation Group (VSIG), Therapeutic Goods Administration (TGA), Australia

[7] Murdoch Children's Research Institute, Parkville, Victoria, Australia

[8] Department of Medicine, The Doherty Institute, The University of Melbourne, Melbourne, Victoria, Australia

[9] Laboratory Services, The Royal Children's Hospital, Parkville, Victoria, Australia

[10] Institute of Infection & Global Health, University of Liverpool, United Kingdom

[11] Department of Paediatrics, The University of Melbourne, Parkville, Victoria, Australia

Keywords: Infectious Disease Medicine, Paediatrics, Technology, Child Health, Health services research

Word count: 2423

Confidential: For Review Only

Abstract

Background: This study aims to describe the use of a paediatric advice line (PAL) provided to parents whose infants were recruited to a large randomised controlled trial, including the number and types of medical concerns addressed, seasonal variability and call outcomes. Additionally, sociodemographic characteristics of the parents and children of those parents who used the PAL are compared with those who did not.

Methods: Prospective cohort of 1246 children nested in the MIS BAIR randomised controlled trial. All MIS BAIR participants were offered access to the PAL. Data was collected over the initial 2 years of a 5-year follow-up. Data was analysed using chi-squared tests, and odds ratios were calculated using multivariate logistic regression.

Results: The PAL was used by 230 (18.5%) participants, who made a total of 586 calls during the 2-year study period. The reasons for calling the PAL were: dermatological (24%); gastrointestinal (18%); disturbances in feeding, sleeping and crying (14%); respiratory (7%); and developmental/neurological (6%). Multivariate analysis revealed that those who used the PAL were more likely to be first-time parents (OR 1.4, 95% CI 1.1 – 1.9) and mothers who hold a university degree (OR 3.3, 95% CI 1.3 – 8.4). PAL costs were minimal and comprised of 15 clinicians with paediatric experience.

Conclusions: A cost-effective PAL service for clinical trial participants was used appropriately by parents for relatively minor concerns, and may have a role in trials to promote participant engagement and reduce demand for other health services.

Confidential: For Review Only

What is already known on this topic

1. Paediatric advice services improve patient satisfaction, help-seeking and informed decision-making.
2. Medical advice hotlines have yet to be studied in paediatric clinical trials.

What this study adds

1. This is the first assessment of the use of a paediatric advice line (PAL) within a clinical trial setting.
2. Parental demand for non-urgent advice within a clinical trial exists for a variety of medical concerns.
3. First-time parents and mothers with a university degree were more likely to use the PAL.

How this study might affect research, practice or policy

1. Advice lines for non-urgent medical concerns offer an economical and valuable means of parental engagement in paediatric clinical trials.

Confidential: For Review Only

Introduction

The Melbourne Infant Study: BCG for Allergy and Infection Reduction (MIS BAIR) is an ongoing, multicentre randomised controlled trial (RCT) to determine the off-target effects of neonatal Bacillus Calmette-Guérin (BCG) vaccination on allergies, eczema, infections and asthma in Australian infants. (1) To provide some benefit to parents of participants of the trial, we established a paediatric advice line (PAL). This MIS BAIR PAL study, a prospective cohort study, was embedded within the MIS BAIR RCT. The intention was to provide access to non-urgent, telephone-based, paediatric medical advice. Here we report the use of the MIS BAIR PAL, including the number and types of medical concerns addressed, seasonal variability, call outcomes and the setup of the PAL. Additionally, we describe the sociodemographic characteristics of those parents who used the PAL compared with those who did not, as well as antenatal, intrapartum and early parenthood factors.

Methods

Study setting and PAL development

In the MIS BAIR trial, 8552 participants were assessed for eligibility with 7280 excluded and 1272 healthy neonates randomised to receive BCG-Denmark vaccine or no vaccine within 10 days of birth (Figure 1). The MIS BAIR eligibility criteria are published open-access in the MIS BAIR study protocol. (1) Infants were followed up regularly for up to five years with parent-completed questionnaires and blood sample collection. (1) A PAL was made available to the parents of all the infants recruited to MIS BAIR for the first two years of their participation as recognition for their contribution to the trial.

The PAL involved a single contact number linked to a mobile phone carried by a clinical staff member during standard business hours (0900 – 1700 on weekdays). Parents were told that it was intended only for non-urgent medical questions and a voicemail could be left by callers outside of standard business hours with a return call within one business day. A clinical member

Confidential: For Review Only

of the study team (doctor, nurse or midwife with paediatric experience) responded to all calls and provided advice (sometimes with emailed resources). The PAL comprised of 15 clinicians rostered to hold the PAL mobile device on a voluntary rotating roster for the duration of one day. If a nurse or midwife receiving PAL calls on a particular day required medical input, a referral was completed to a medical MIS BAIR staff member with a return call within one business day. Parents calling for a rash or skin condition were asked to send photographs by email or smartphone. If appropriate, follow-up calls were arranged with a nurse, a paediatrician, the participant's general practitioner, a specialist clinic or at the emergency department.

Data collection

Details of all calls were recorded by the clinical member answering the call using a Research Electronic Data Capture (REDCap) database, including principal concern(s) and outcome of the call. Follow-up calls were recorded in chronological order and linked to the same participant number. The data were subsequently cleaned, categorised and coded in a standardised manner by JC and JK.

Definitions

A PAL caller was defined as a parent who remained in the trial for at least 3 months (completed at least one of the 3-monthly questionnaires) and who called the PAL at least once during the first 2 years of their participation in the trial. A PAL non-caller was defined as a parent who remained in the trial for at least 3 months (completed at least one of the 3-monthly questionnaires) but who did not call the PAL. Parents who called exclusively with questions about the trial (including BCG vaccine injection site concerns) were defined as non-callers, and these calls were not included in the analysis. The principal concern was determined as the initial symptom discussed during the call, with other symptoms listed as additional concerns.

Statistical analysis

Confidential: For Review Only

The sociodemographic characteristics of PAL callers were compared with non-callers using chi-square tests, Fisher's exact tests, independent samples t-tests and Wilcoxon-Mann-Whitney tests. Univariate logistic regression was used to identify independent factors associated with the use of the PAL. All significant predictors ($p < 0.20$) were then included in a stepwise backward multivariate analysis. Statistical analysis was done using Stata version 16.

Ethics approval

The PAL was included in the MIS BAIR ethical and governance approval from Mercy Health Human Research Ethics Committee (HREC, No. R12-28) and Royal Children's Hospital HREC (No. 33025) with additional governance approval from Barwon Health and St John of God, Geelong, Victoria. Signed informed consent was obtained from the parents of all participants.

Patient and Public Involvement Statement

The MIS BAIR PAL was developed with the interests of the public and the MIS BAIR participants at the forefront of the study design. The primary outcome was to develop a PAL and observe parental use with the aim of improving patient and parental engagement. Patients were not directly involved in the development of the study design and future analyses aim to further investigate parental experience and satisfaction with the PAL.

Results

A total of 1246 participants were included in the final analysis and 26 were lost to follow-up after randomisation (2.0%) (Figure 1). The demographics of the study population are detailed in Table 1. There was an approximately even distribution of sex of the infant and their BCG vaccination status. Most participants were born via vaginal delivery, and most were breastfed.

A total of 586 calls were recorded during the study. A total of 230 parents (18.5%) called the PAL at least once during the first 2 years of their participation in the trial. Of these, 69 (30%)

Confidential: For Review Only

contacted the PAL on only one occasion (inclusive of subsequent follow-up calls) and 161 (70%) on multiple occasions.

Principal concern for call

Figures 2 and 3 illustrate the age distribution and the principal concerns for the PAL calls. The most common reason for a call was a rash or skin condition (87 calls), with calls distributed equally across infancy. The second most common reason was for a concern relating to feeding, sleeping or crying (47 calls), with most occurring before 20 weeks of age. A total of 34 and 29 calls were related to changes in bowel habit or vomiting, respectively, with most calls received during the neonatal period. Calls related to respiratory symptoms (26 calls) had a bi-modal presentation, clustered around 10 and 60 weeks of age. A total of 12 calls were for fever. Of the 87 dermatological-related calls, 3 were associated with a fever. Of the 26 respiratory symptom-related calls, 4 were associated with a fever. Developmental or neurological concerns (21 calls) occurred in early infancy, as did growth concerns (4 calls). Calls listed as "Other medical concern – baby unwell" included 32 calls for various concerns including ear/nose/throat issues (12 calls), abnormal pathology results or medication queries. A total of 37 calls were for general advice, mainly related to travel (9 calls), obtaining vaccines (12 calls), and introduction of solids to the infant's diet (12 calls).

Seasonal variability

There was seasonal variability in PAL calls for all of the principal concerns (Figure 4). Calls for rashes and other skin conditions occurred more commonly during summer and winter. Calls related to feeding, sleeping, crying, changes of bowel habit, vomiting or respiratory symptoms occurred more commonly during autumn and winter. Travel advice was sought most commonly during winter. Participant recruitment was approximately evenly distributed across all seasons, accounted for as a potential confounding factor, shown in Appendix A.

Call outcomes

Confidential: For Review Only

Of the 586 calls to the PAL, 314 (53.6%) of the parents' concern(s) were resolved in a single call, 185 (31.7%) in 2 calls, and 87 (14.8%) in 3 or more calls. For the 268 calls for which this information was available, the outcomes were verbal medical advice given by a paediatrician (141 calls, 52.6%), verbal advice from a paediatric nurse or midwife (60 calls, 22.4%), written advice from a PAL clinician (email or text message) (18 calls, 6.7%), referral to be seen in-person by a paediatrician (11 calls, 4.1%), and referral to a general practitioner (31 calls, 11.6%) or the emergency department (7 calls, 2.6%). Advice was either given during the initial call or shortly after if advice from a more senior clinician was required.

Sociodemographics of PAL callers

The sociodemographic characteristics of PAL callers and non-callers were compared (Table 1). PAL callers had a slightly higher maternal age (mean 33.3 years, SD 4.1) compared with non-callers (mean 32.6 years, SD 4.9, p=0.06). First-time parents were more likely to call the PAL than non-first-time parents (140/649 (21.6%) vs. 90/597 (15.1%), p=0.003). Parents of infants conceived though IVF or donor gametes were also more likely to call (8/20 (40%) vs. 49/333 (14.7%), p=0.003), as were parents of infants who were breastfed (187/927 (20.2%) vs. 2/37 (5.4%), p=0.03) and whose mothers were university-educated (185/848 (22%) vs. 44/395 (11%), p<0.001). There were no statistically significant differences between the PAL caller and non-caller groups in relation to sex of the infant, mode of delivery, birthweight, maternal birthplace, or BCG vaccination status.

Multivariate analysis

Univariate and multivariate analyses are outlined in Table 2. In the final multivariate analysis, being a first-time parent remained a statistically significant predictor for calling the PAL (OR 1.4, 95% CI 1.1 – 1.9), as did maternal attainment of a university degree (OR 3.3, 95% CI 1.3 – 8.4). Breastfeeding and higher maternal age were not retained in the final multivariate analysis as they were no longer predicting the outcome, likely due to them being confounded by higher maternal education status in the model.

Confidential: For Review Only

Confidential: For Review Only

Discussion

Paediatric advice services provide guidance for parents, enhance clinician-patient communication, and improve parental satisfaction and accessibility to non-urgent health advice. (2-4) To our knowledge, the MIS BAIR PAL is among the first paediatric advice services for parents associated with a large RCT. Given approximately 1 in 5 parents used the PAL, with a high proportion of parents calling multiple times, this highlights a demand for non-urgent medical advice during early childhood. Whilst studies have shown a growing demand for medical triage and assessment hotlines, this has not been studied in a clinical trial setting. (5)

The PAL operated via a single mobile phone attended by a clinical staff member during standard business hours on a voluntary rotating roster basis. The overall cost was minimal, with one-off costs for a mobile device and telephone number, given the voluntary basis of the team and the operation during business hours. Given 46.4% of PAL calls required a follow-up consultation, continuity of care was one challenge faced by PAL clinicians. However, detailed clinical documentation with all follow-up consultations occurring within one business day allowed for a streamlined process. Telephone consultation without real-time visualisation of a dermatological complaint provided some difficulty in clinical assessment. Photographs provided by parents assisted in clinical decision-making. However, using a live audio-visual telehealth service in future studies may ameliorate this challenge.

In our study, the majority of concerns could be addressed by PAL staff alone and the large majority of concerns were resolved within 2 telephone calls. This is likely to have reduced the demand on emergency services for non-urgent concerns. Factors including the parent's perception of urgency, uncertainty in their own assessment of the child's symptoms and unawareness of service availability all influence a parent's decision to seek help. (6) Providing a

PAL for non-urgent concerns provides an accessible means for parents to understand the importance of their child's symptoms and better inform parental decision-making in relation to whether they need to seek further help.

Dermatological concerns were the most common reason to contact the PAL across all ages, with a proclivity for summer and winter months. Many dermatological conditions, such as miliaria, atopic dermatitis and viral exanthems, present frequently during early childhood, often with seasonal variability. (7-9) Calls for respiratory concerns, gastrointestinal symptoms and disturbances with sleeping, feeding and crying were all clustered within the first 20 weeks of life; this is most likely due to early parenthood anxiety, given the high prevalence of first-time and breastfeeding parents amongst the PAL callers. The increased frequency of these calls during the winter months most likely correlates with higher incidence rates of bronchiolitis, croup and viral infections, with associated gastrointestinal upset and irritability. (10, 11) The clustering of "other" medical concerns in well infants around 30 weeks of age may correspond with the introduction of solids into the diet, often a time of apprehension. (12) Only a small proportion of calls were related to febrile episodes, even accounting for fever listed as an associated symptom rather than the main presenting concern. This may be due to parents presenting to a medical setting directly, recognising it as a potential emergency. (13)

PAL callers were more likely to be older mothers, first-time parents, university-educated and/or have used IVF or donor gametes compared to non-callers. This is consistent with literature indicating that first-time mothers of a higher maternal age have increased levels of psychological stress and anxiety during both pregnancy and the post-partum period. (14) Pregnancies acquired with the use of IVF and oocyte/sperm donation are more likely to develop stress and anxiety, (17) partly as a result of the greater risk of antenatal and intra-partum complications. (15, 16) Mothers with a university education might also feel more empowered to ask for advice than those without or those with a language barrier. Further investigation of the influence of other socioeconomic factors, such as health literacy, income and employment

Confidential: For Review Only

status, on the willingness to access and use the advice line is required as this study suggests lower educational attainment may be a barrier to PAL use.

Whilst patient satisfaction is associated with the availability and use of medical advice lines, (4) a limitation of our study is the lack of data on the influence of the PAL availability on parents' willingness to take part in the MIS BAIR trial, their satisfaction and retention rates. Future studies should address whether advice lines provide an effective incentive to improve trial participation and/or retention, and seek parents' feedback and evaluation of their benefit. Questionnaires and cluster randomised cohort studies may be of use. Additionally, our results may not be generalisable as the parents, having self-selected to participate in a trial, may not be representative of the wider population. Future PAL services could be improved by having a pre-planned referral pathway and information brochure available for common parental concerns.

Paediatric advice services are well-recognised as valuable and convenient means of empowering parents and reducing parental concerns, helping to inform decision-making and parental help-seeking. (4) The MIS BAIR PAL study demonstrates that demand for such a service exists within a clinical trial setting and is likely to be used by parents during early parenthood. Additionally, this prospective cohort study, embedded within a host RCT, adds to the evidence base for trialists conducting a Study Within A Trial (SWAT), informing design and conduct of future trials. (18)

Confidential: For Review Only

Funding:

The MIS BAIR trial is funded by research grants from the National Health and Medical Research Council of Australia (GNT1051228 and GNT1099676), The University of Melbourne, The Royal Children's Hospital Foundation, the Murdoch Children's Research Institute, Mercy Health, the Myers Foundation, the European Society of Pediatric Infectious Diseases, the Nossal Institute for Global Health, and the Aranday Foundation. Dr Laure Pittet is supported by the Swiss National Science Foundation (Early Postdoc Mobility Grant number P2GEP3_178155).

Contribution Statement:

JC and JK contributed equally to this paper and are joint first authors. JC and JK transcribed the call data collected, performed statistical analysis and wrote the manuscript. NC and LP contributed equally to this paper and are joint last authors. NC and LP were integral to the study funding, design and manuscript production. All other authors were integral in data collection and management of the paediatric advice line, including taking calls and providing advice where necessary.

Acknowledgments:

The authors thank the MIS BAIR participants and their families for their involvement in the trial. We also thank our recruitment and follow-up teams.

Conflict of Interest:

There are no reportable conflicts of interest.

Confidential: For Review Only

References:

1. Messina NL, Gardiner K, Donath S, Flanagan K, Ponsonby AL, Shann F et al. Study protocol for the Melbourne Infant Study: BCG for Allergy and Infection Reduction (MIS BAIR), a randomised controlled trial to determine the non-specific effects of neonatal BCG vaccination in a low-mortality setting. BMJ open. 2019 Dec 1;9(12):e032844.
2. Wang CJ, Ma J, Zuckerman B, Car J. The Opportunities for Telehealth in Pediatric Practice and Public Health. Pediatr Clin North Am. 2020 Aug;67(4):603-611. doi: 10.1016/j.pcl.2020.03.001.
3. Siddiqui, N., D. Greenfield and A. Lawler. Calling for confirmation, reassurance, and direction: Investigating patient compliance after accessing a telephone triage advice service. Int. J. Health Plan. Manag. 2020 May;35(3): 735-745. DOI: https://dx.doi.org/10.1002/hpm.2934
4. Li L, Georgiou A, Xiong J, Byrne M, Robinson M, Westbrook JI. Healthdirect's After Hours GP Helpline-A Survey of Patient Satisfaction with the Service and Compliance with Advice. InHIC 2016 Jan 1 (pp. 87-92).
5. Gibson A, Randall D, Tran DT, Byrne M, Lawler A, Havard A et al. Emergency Department Attendance after Telephone Triage: A Population-Based Data Linkage Study. Health services research. 2018 Apr;53(2):1137-62.
6. McLauchlan K, Ramlakhan S, Irving A. Why do parents present to the Paediatric Emergency Department with conditions suitable for management in less acute settings? A qualitative study. Eur J Emerg Med. 2020 Feb 1;27(1):40-5.
7. Kutlubay Z, Tanakol A, Engyn B, Onel C, Symsek E, Serdaroglu S et al. Newborn skin: common skin problems. Maedica. 2017 Jan;12(1):42.
8. Foley P, Zuo Y, Plunkett A, Marks R. The frequency of common skin conditions in preschool-age children in Australia: atopic dermatitis. Arch. Dermatol. 2001 Mar 1;137(3):293-300.

Confidential: For Review Only

9. Foley P, Zuo Y, Plunkett A, Merlin K, Marks R. The frequency of common skin conditions in preschool-aged children in Australia: seborrheic dermatitis and pityriasis capitis (cradle cap). Arch. Dermatol. 2003 Mar 1;139(3):318-22.
10. Lipsett SC, Monuteaux MC, Fine AM. Seasonality of common pediatric infectious diseases. Pediatr Emerg Care. 2021 Feb 1;37(2):82-5.
11. Hoeppner T, Borland M, Babl FE, Neutze J, Phillips N, Krieser D et al. Influence of weather on incidence of bronchiolitis in Australia and New Zealand. J Paediatr Child. 2017 Oct;53(10):1000-6.
12. Arora A, Manohar N, Hector D, Bhole S, Hayen A, Eastwood J et al. Determinants for early introduction of complementary foods in Australian infants: findings from the HSHK birth cohort study. Nutrition journal. 2020 Dec;19(1):1-0.
13. Bereznicki BJ, Tucker MG, Beggs SA, Zosky GR, Bereznicki LR. Emergency department presentations of febrile children to an Australian public hospital. J Paediatr Child. 2018 Dec;54(12):1308-13.
14. Aasheim V, Waldenström U, Hjelmstedt A, Rasmussen S, Pettersson H, Schytt E. Associations between advanced maternal age and psychological distress in primiparous women, from early pregnancy to 18 months postpartum. BJOG BJOG-INT J OBSTET GY. 2012 Aug;119(9):1108-16.
15. Berntsen S, Larsen EC, la Cour Freiesleben N, Pinborg A. Pregnancy outcomes following oocyte donation. Best Pract. 2021 Jan 1;70:81-91.
16. Wertheimer A, Melamed S, Ashwal E, Sapir O, Oron G, Ben-Haroush A et al. Complications of the third stage of labor are more prevalent in IVF pregnancies. J. Matern. 2020 Feb 21:1-5.
17. Hammarberg K, Fisher JR, Wynter KH. Psychological and social aspects of pregnancy, childbirth and early parenting after assisted conception: a systematic review. Hum. Reprod. 2008 Sep 1;14(5):395-414.
18. Treweek S, Bevan S, Bower P, Campbell M, Christie J, Clarke M, Collett C, Cotton S, Devane D, El Feky A, Flemyng E. Trial forge guidance 1: what is a study within a trial (SWAT)?. Trials. 2018 Dec;19(1):1-5.

Confidential: For Review Only

Table 1. PAL study parent and infant demographics.

	All participants (n=1246)	PAL callers (n=230)	PAL non-callers (n=1016)	P-value
**Sex of child**				
**Male**	628 (50.4%)	117 (50.9%)	511 (50.3%)	0.9
**Female**	618 (49.6%)	113 (49.1%)	505 (49.7%)	
**First time parent**				
**Yes**	649 (52.1%)	140 (60.9%)	509 (50.1%)	0.003
**No**	597 (47.9%)	90 (39.1%)	507 (49.9%	
**Number of siblings**				
**0**	649 (52.1%)	140 (60.9%)	509 (50.1%)	0.02
**1**	388 (31.1%)	69 (30.0%)	319 (31.4%)	
**2**	150 (12.0%)	15 (6.5%)	135 (13.3%)	
**3**	34 (2.7%)	4 (1.7%)	30 (3.0%)	
**4**	13 (1.0%)	1 (0.4%)	12 (1.2%)	
**5**	10 (0.8%)	1 (0.4%)	9 (0.9%)	
**6**	0 (0.0%)	0 (0.0%)	0 (0.0%)	
**7**	2 (0.2%)	0 (0.0%)	2 (0.2%)	
**Birth method**				
**Vaginal**	800 (64.2%)	150 (65.2%)	650 (64.0%)	0.7
**Caesarean**	446 (35.8%)	80 (34.8%)	366 (36.0%)	
**Gestational age**				
**Weeks***	39.3 (38.4, 40.3)	39.4 (38.5, 40.5)	39.3 (38.4, 40.3)	0.1
**IVF or sperm/egg donor**				
**IVF/donor used**	20 (1.6%)	8 (3.5%)	12 (1.2%)	0.003
**No IVF/donor used**	333 (26.7%)	49 (21.3%)	284 (28.0%)	
**Unknown**	893 (71.7%)	173 (75.2%)	720 (70.9%)	
**Birthweight**				
**Birthweight (kg)***	3.4 (3.1, 3.7)	3.4 (3.1, 3.8)	3.4 (3.1, 3.7)	0.3
**Maternal age at delivery**				

Confidential: For Review Only

Years#	32.7 (4.7)	33.3 (4.1)	32.6 (4.9)	0.06
**Mother's birthplace**				
Australia	902 (72.4%)	169 (73.5%)	733 (72.2%)	0.2
Outside Australia	344 (27.6%)	61 (26.5%)	283 (27.9%)	
**Maternal education**				
No education/up to Year 10	69 (5.5%)	5 (2.2%)	64 (6.3%)	<0.001
Year 12/trade	326 (26.2%)	39 (17.0%)	287 (28.1%)	
University	848 (68.1%)	185 (80.4%)	663 (65.3%)	
Unknown	3 (0.2%)	1 (0.4%)	2 (0.2%)	
**Breastfed**				
Breastfed	927 (74.4%)	187 (81.3%)	740 (72.8%)	0.03
Not breastfed	37 (3.0%)	2 (0.9%)	35 (3.4%)	
Unknown	282 (22.6%)	41 (17.8%)	241 (23.7%)	
**Received BCG**				
Yes	622 (49.9%)	119 (51.7%)	503 (49.5%)	0.4
No	624 (50.1%)	111 (48.3%)	513 (50.5%)	

Reported as number (%) and p-value calculated using chi-square or Fisher's exact test, unless otherwise noted.

* Reported as median (IQR). P-value calculated using Wilcoxon-Mann-Whitney test.

# Reported as mean (SD). P-value calculated using independent samples t-test.

Confidential: For Review Only

Confidential: For Review Only

Table 2. Univariate and multivariate analyses predicting PAL callers.

	Univariate model OR* (95% CI)	P-value	Multivariate model OR* (95% CI)	P-value
**Sex**				
**Male**	1.0 (0.8 – 1.4)	0.9		
**First time parent**				
**Yes**	1.5 (1.2 – 2.1)	0.003	1.4 (1.1 – 1.9)	0.02
**Number of siblings**				
**0**	Reference	Reference		
**1**	0.8 (0.6 – 1.1)	0.1		
**2**	0.4 (0.2 – 0.7)	0.002		
**3**	0.5 (0.2 – 1.4)	0.2		
**4**	0.3 (0.04 – 2.4)	0.3		
**5**	0.4 (0.05 – 3.2)	0.4		
**Birth method**				
**Vaginal**	1.1 (0.8 – 1.4)	0.7		
**Gestational age**				
**Weeks**	1.1 (1.0 – 1.2)	0.08		
**IVF or sperm/egg donor**				
**Yes**	3.9 (1.5 – 9.9)	0.005#		
**Birthweight**				
**<2500 g**	0.8 (0.3 – 1.8)	0.5		
**Birthweight (kg)**	1.2 (0.9 – 1.6)	0.2		
**Maternal age at delivery**				
**Years**	1.0 (1.0 – 1.1)	0.06		
**Mother's birthplace**				
**Australia**	1.1 (0.8 – 1.5)	0.7		
**Maternal education**				
**No education/up to Year 10**	Reference	Reference	Reference	
**Year 12/trade**	1.7 (0.7 – 4.6)	0.3	1.7 (0.6 – 4.5)	0.3
**University**	3.6 (1.4 – 9.0)	0.007	3.3 (1.3 – 8.4)	0.01

Confidential: For Review Only

**Breastfed**		
**Yes**	4.4 (1.1 – 18.6)	0.04
**Received BCG**		
**Yes**	1.1 (0.8 – 1.5)	0.5

* Odds ratio calculated using logistic regression method.

IVF was not included in the multivariate analysis due to missing data resulting in loss of statistical power.

Confidential: For Review Only

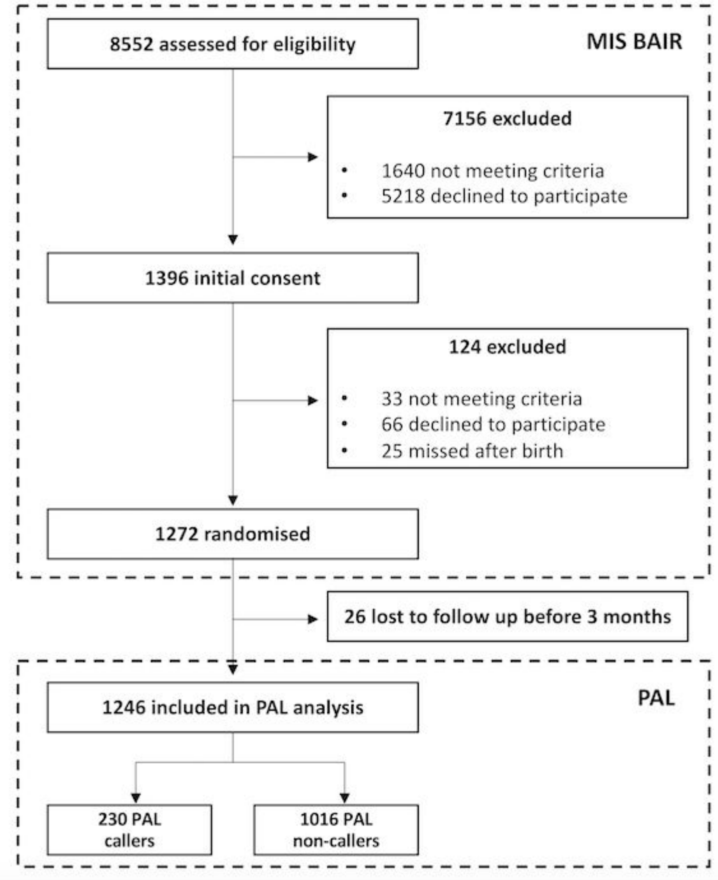

Figure 1. Consort diagram for the MIS BAIR PAL participants.

For Peer Review Only

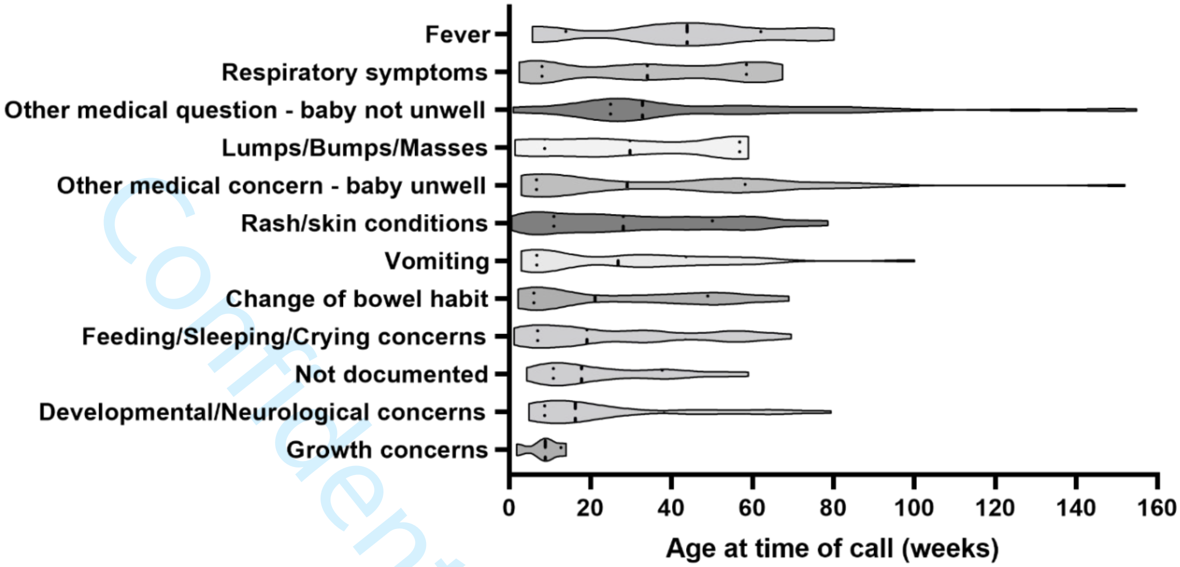

Figure 2. Age distribution of infants at the time of the parent call to the PAL categorised by reasons.

Confidential: For Review Only

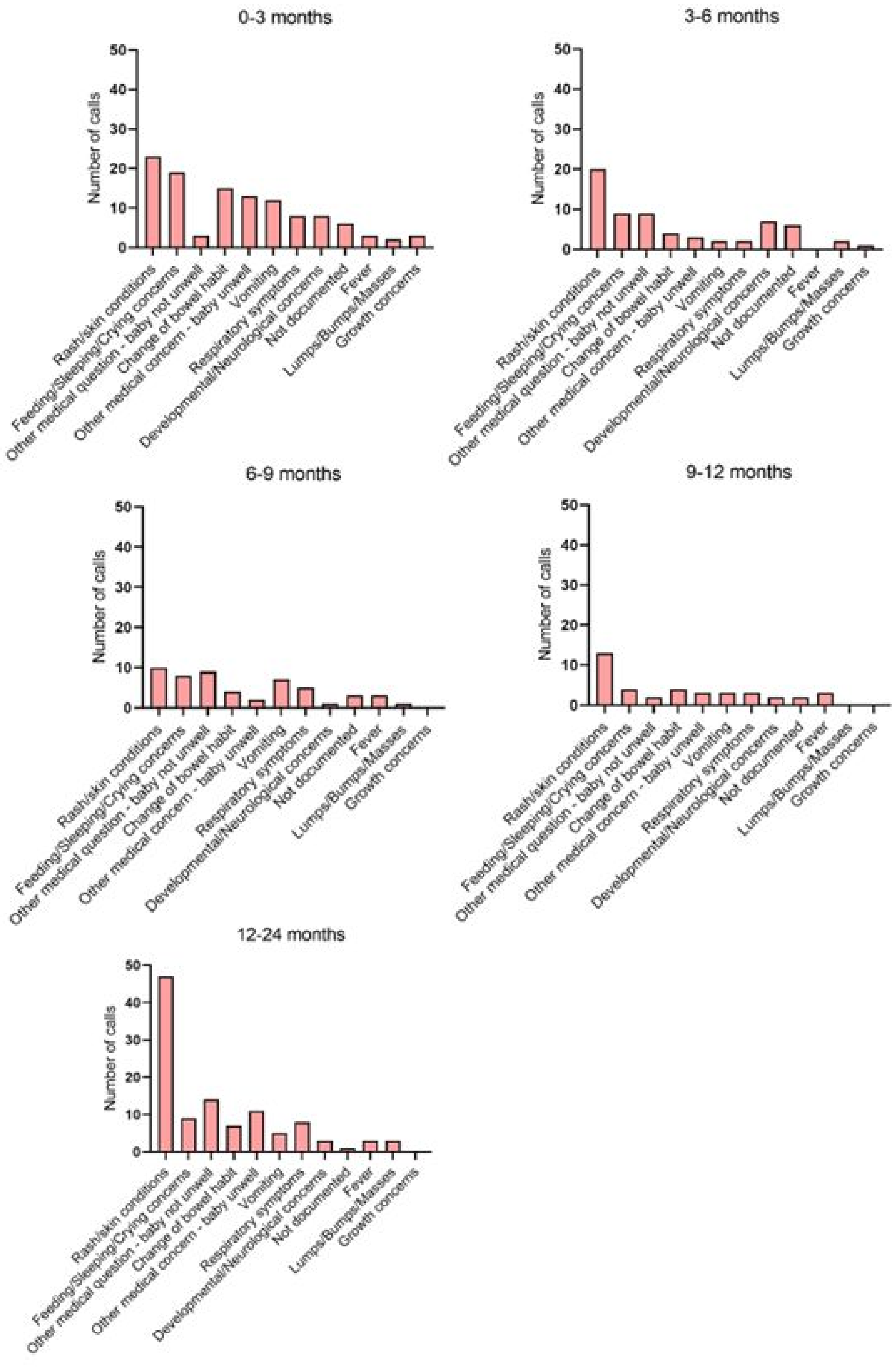
0-3 months
Number of calls
0
10
20
30
40
50
Rash/skin conditions
Feeding/Sleeping/Crying concerns
Other medical question - baby not unwell
Change of bowel habit
Other medical concern - baby unwell
Vomiting
Respiratory symptoms
Developmental/Neurological concerns
Not documented
Fever
Lumps/Bumps/Masses
Growth concerns
3-6 months
Number of calls
0
10
20
30
40
50
Rash/skin conditions
Feeding/Sleeping/Crying concerns
Other medical question - baby not unwell
Change of bowel habit
Other medical concern - baby unwell
Vomiting
Respiratory symptoms
Developmental/Neurological concerns
Not documented
Fever
Lumps/Bumps/Masses
Growth concerns
6-9 months
Number of calls
0
10
20
30
40
50
Rash/skin conditions
Feeding/Sleeping/Crying concerns
Other medical question - baby not unwell
Change of bowel habit
Other medical concern - baby unwell
Vomiting
Respiratory symptoms
Developmental/Neurological concerns
Not documented
Fever
Lumps/Bumps/Masses
Growth concerns
9-12 months
Number of calls
0
10
20
30
40
50
Rash/skin conditions
Feeding/Sleeping/Crying concerns
Other medical question - baby not unwell
Change of bowel habit
Other medical concern - baby unwell
Vomiting
Respiratory symptoms
Developmental/Neurological concerns
Not documented
Fever
Lumps/Bumps/Masses
Growth concerns
12-24 months
Number of calls
0
10
20
30
40
50
Rash/skin conditions
Feeding/Sleeping/Crying concerns
Other medical question - baby not unwell
Change of bowel habit
Other medical concern - baby unwell
Vomiting
Respiratory symptoms
Developmental/Neurological concerns
Not documented
Fever
Lumps/Bumps/Masses
Growth concerns

Figure 3. Frequency of PAL calls for each reason at different infant ages.

Confidential: For Review Only

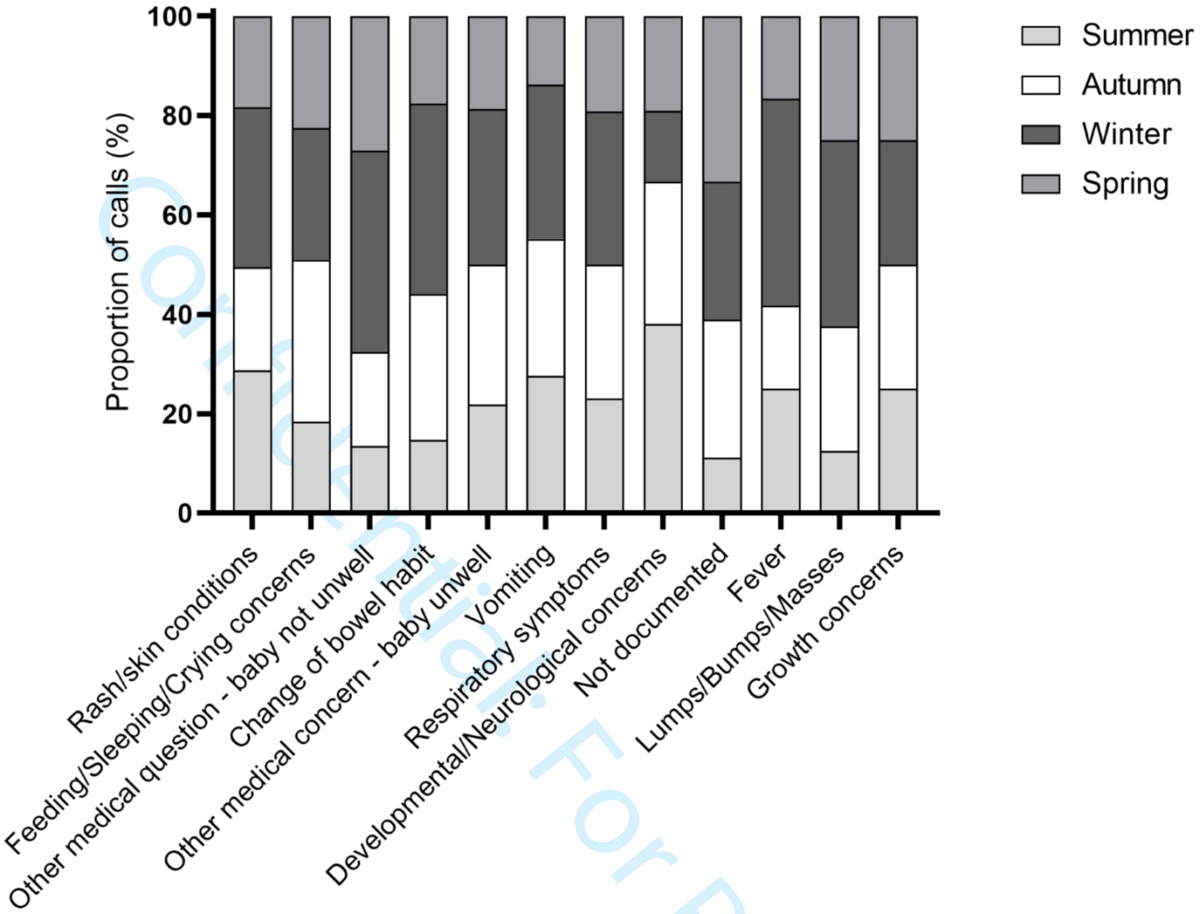

Figure 4. Seasonal distribution of calls to the PAL for each reason during the first 2 years of life.

Confidential: For Review Only

Appendix A. Participant recruitment timing based on date of birth in 3-monthly periods.

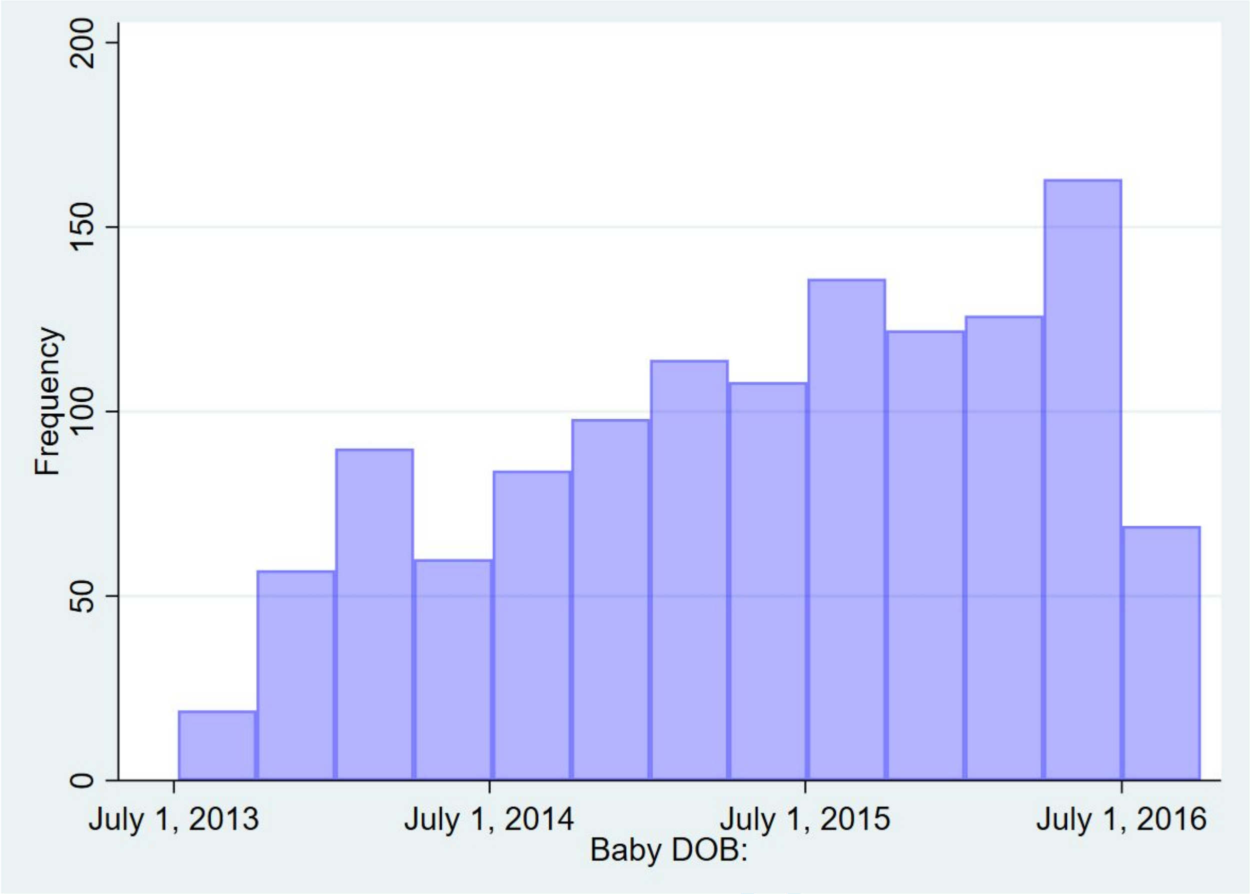

For peer review Only

BMJ Paediatrics Open

Use of a paediatric advice line for parents of infants recruited to a randomised controlled trial

Journal:	*BMJ Paediatrics Open*
Manuscript ID	bmjpo-2022-001665.R2
Article Type:	Original research
Date Submitted by the Author:	04-Nov-2022
Complete List of Authors:	Chan, Joyce; Murdoch Children's Research Institute, Infectious Diseases Research Group; The Royal Melbourne Hospital<br>Kirby, Jordan; Mercy Hospital for Women, Department of Obstetrics & Gynaecology<br>Gardiner, Kaya; Murdoch Children's Research Institute, Infectious Diseases Research Group<br>Abruzzo, Veronica; Murdoch Children's Research Institute, Infectious Diseases Research Group<br>Bannister, Samantha; Murdoch Children's Research Institute, Infectious Diseases Research Group; Royal Children's Hospital Research Institute, Infectious Diseases Unit<br>Bellamy, Karen; Australian Technical Advisory Group on Immunisation (ATAGI); Vaccine Safety Investigation Group (VSIG)<br>Brophy, Clare; Murdoch Children's Research Institute; The Peter Doherty Institute for Infection and Immunity<br>Clifford, Vanessa; Royal Children's Hospital Research Institute, Infectious Diseases Unit; Royal Children's Hospital Research Institute, Laboratory Services<br>Cox, Lianne; Murdoch Children's Research Institute; Royal Children's Hospital Research Institute,<br>Elborough, Hannah; Murdoch Children's Research Institute<br>Freyne, Bridget Freyne; University of Liverpool, Institute of Infection & Global Health<br>Summons, Freya; Murdoch Children's Research Institute, Infectious Diseases Research Group; The University of Melbourne, Department of Paediatrics<br>Morrison, Clare; Murdoch Children's Research Institute<br>Wall, Kate; Murdoch Children's Research Institute<br>Casalaz, Daniel; Mercy Hospital for Women, Department of Paediatrics<br>Curtis, Nigel; The University of Melbourne, Department of Paediatrics; Murdoch Childrens Research Institute, Infectious Diseases Research Group<br>Pittet, Laure F.; Murdoch Children's Research Institute, Infectious Diseases Research Group; The University of Melbourne, Department of Paediatrics
Keywords:	Technology, Health services research

I, the Submitting Author has the right to grant and does grant on behalf of all authors of the Work (as defined in the below author licence), an exclusive licence and/or a non-exclusive licence for contributions from authors who are: i) UK Crown employees; ii) where BMJ has agreed a CC-BY licence shall apply, and/or iii) in accordance with the terms applicable for US Federal Government officers or employees acting as part of their official duties; on a worldwide, perpetual, irrevocable, royalty-free basis to BMJ Publishing Group Ltd ("BMJ") its licensees and where the relevant Journal is co-owned by BMJ to the co-owners of the Journal, to publish the Work in this journal and any other BMJ products and to exploit all rights, as set out in our licence.

The Submitting Author accepts and understands that any supply made under these terms is made by BMJ to the Submitting Author unless you are acting as an employee on behalf of your employer or a postgraduate student of an affiliated institution which is paying any applicable article publishing charge ("APC") for Open Access articles. Where the Submitting Author wishes to make the Work available on an Open Access basis (and intends to pay the relevant APC), the terms of reuse of such Open Access shall be governed by a Creative Commons licence – details of these licences and which Creative Commons licence will apply to this Work are set out in our licence referred to above.

Other than as permitted in any relevant BMJ Author's Self Archiving Policies, I confirm this Work has not been accepted for publication elsewhere, is not being considered for publication elsewhere and does not duplicate material already published. I confirm all authors consent to publication of this Work and authorise the granting of this licence.

Confidential: For Review Only

Use of a paediatric advice line for parents of infants recruited to a randomised controlled trial

Corresponding Author: Dr Joyce Chan (Murdoch's Children's Research Institute, 50 Flemington Rd, Parkville VIC 3052. Email: joyce.chan@mcri.com.au Telephone: (03) 8341 6200

Authors:

Joyce Chan [1, 2], Jordan Kirby [3] (joint first authors), Kaya Gardiner [1], Veronica Abruzzo [1], Samantha Bannister [1, 4], Karen Bellamy [5, 6], Clare Brophy [7,] 8, Vanessa Clifford [4, 9], Lianne Cox [7], Hannah Elborough [7], Bridget Freyne [10], Freya Summons [1, 11], Clare Morrison [7], Kate Wall[7], Dan Casalaz [3], Nigel Curtis [1, 11], Laure F Pittet [1, 11] (joint last authors) on behalf of the MIS BAIR trial group

[1] Infectious Diseases Research Group, Murdoch Children's Research Institute, Parkville, Victoria, Australia

[2] The Royal Melbourne Hospital, Parkville, Victoria, Australia

[3] Mercy Hospital for Women, Heidelberg, Victoria, Australia

[4] Infectious Diseases Unit, The Royal Children's Hospital, Parkville, Victoria, Australia

[5] Australian Technical Advisory Group on Immunisation (ATAGI), Australia

[6] Vaccine Safety Investigation Group (VSIG), Therapeutic Goods Administration (TGA), Australia

[7] Murdoch Children's Research Institute, Parkville, Victoria, Australia

[8] Department of Medicine, The Doherty Institute, The University of Melbourne, Melbourne, Victoria, Australia

[9] Laboratory Services, The Royal Children's Hospital, Parkville, Victoria, Australia

[10] Institute of Infection & Global Health, University of Liverpool, United Kingdom

[11] Department of Paediatrics, The University of Melbourne, Parkville, Victoria, Australia

Keywords: Infectious Disease Medicine, Paediatrics, Technology, Child Health, Health services research

Word count: 2423

Confidential: For Review Only

Abstract

Background: This study aims to describe the use of a paediatric advice line (PAL) provided to parents whose infants were recruited to a large randomised controlled trial, including the number and types of medical concerns addressed, seasonal variability and call outcomes. Additionally, sociodemographic characteristics of the parents and children of those parents who used the PAL are compared with those who did not.

Methods: Prospective cohort of 1246 children nested in the MIS BAIR randomised controlled trial. All MIS BAIR participants were offered access to the PAL. Data was collected over the initial 2 years of a 5-year follow-up. Data was analysed using chi-squared tests, and odds ratios were calculated using multiple logistic regression.

Results: The PAL was used by 230 (18.5%) participants, who made a total of 586 calls during the 2-year study period. The reasons for calling the PAL were: dermatological (24%); gastrointestinal (18%); disturbances in feeding, sleeping and crying (14%); respiratory (7%); and developmental/neurological (6%). Analysis revealed that those who used the PAL were more likely to be first-time parents (OR 1.4, 95% CI 1.1 – 1.9) and mothers who hold a university degree (OR 3.3, 95% CI 1.3 – 8.4). PAL costs were minimal and comprised of 15 clinicians with paediatric experience.

Conclusions: A cost-effective PAL service for clinical trial participants was used appropriately by parents for relatively minor concerns, and may have a role in trials to promote participant engagement and reduce demand for other health services.

Confidential: For Review Only

What is already known on this topic

1. Paediatric advice services improve patient satisfaction, help-seeking and informed decision-making.
2. Medical advice hotlines have yet to be studied in paediatric clinical trials.

What this study adds

1. Parental demand for non-urgent advice within a clinical trial exists for a variety of medical concerns.
1. First-time parents and mothers with a university degree were more likely to use the PAL.

How this study might affect research, practice or policy

1. Advice lines for non-urgent medical concerns offer an economical and valuable means of parental engagement in paediatric clinical trials.

Confidential: For Review Only

Introduction

The Melbourne Infant Study: BCG for Allergy and Infection Reduction (MIS BAIR) is an ongoing, multicentre randomised controlled trial (RCT) to determine the off-target effects of neonatal Bacillus Calmette-Guérin (BCG) vaccination on allergies, eczema, infections and asthma in Australian infants. (1) To provide some benefit to parents of participants of the trial, we established a paediatric advice line (PAL). This MIS BAIR PAL study, a prospective cohort study, was embedded within the MIS BAIR RCT. The intention was to provide access to non-urgent, telephone-based, paediatric medical advice. Here we report the use of the MIS BAIR PAL, including the number and types of medical concerns addressed, seasonal variability, call outcomes and the setup of the PAL. Additionally, we describe the sociodemographic characteristics of those parents who used the PAL compared with those who did not, as well as antenatal, intrapartum and early parenthood factors.

Methods

Study setting and PAL development

In the MIS BAIR trial, 8552 participants were assessed for eligibility with 7280 excluded and 1272 healthy neonates randomised to receive BCG-Denmark vaccine or no vaccine within 10 days of birth (Figure 1). The MIS BAIR eligibility criteria are published open-access in the MIS BAIR study protocol. (1) Infants were followed up regularly for up to five years with parent-completed questionnaires and blood sample collection. (1) A PAL was made available to the parents of all the infants recruited to MIS BAIR for the first two years of their participation as recognition for their contribution to the trial.

The PAL involved a single contact number linked to a mobile phone carried by a clinical staff member during standard business hours (0900 – 1700 on weekdays). Parents were told that it was intended only for non-urgent medical questions and a voicemail could be left by callers outside of standard business hours with a return call within one business day. A clinical member

Confidential: For Review Only

of the study team (doctor, nurse or midwife with paediatric experience) responded to all calls and provided advice (sometimes with emailed resources). The PAL comprised of 15 clinicians rostered to hold the PAL mobile device on a voluntary rotating roster for the duration of one day. If a nurse or midwife receiving PAL calls on a particular day required medical input, a referral was completed to a medical MIS BAIR staff member with a return call within one business day. Parents calling for a rash or skin condition were asked to send photographs by email or smartphone. If appropriate, follow-up calls were arranged with a nurse, a paediatrician, the participant's general practitioner, a specialist clinic or at the emergency department.

Data collection

Details of all calls were recorded by the clinical member answering the call using a Research Electronic Data Capture (REDCap) database, including principal concern(s) and outcome of the call. Follow-up calls were recorded in chronological order and linked to the same participant number. The data were subsequently cleaned, categorised and coded in a standardised manner by JC and JK.

Definitions

A PAL caller was defined as a parent who remained in the trial for at least 3 months (completed at least one of the 3-monthly questionnaires) and who called the PAL at least once during the first 2 years of their participation in the trial. A PAL non-caller was defined as a parent who remained in the trial for at least 3 months (completed at least one of the 3-monthly questionnaires) but who did not call the PAL. Parents who called exclusively with questions about the trial (including BCG vaccine injection site concerns) were defined as non-callers, and these calls were not included in the analysis. The principal concern was determined as the initial symptom discussed during the call, with other symptoms listed as additional concerns.

Statistical analysis

Confidential: For Review Only

The sociodemographic characteristics of PAL callers were compared with non-callers using chi-square tests, Fisher's exact tests, independent samples t-tests and Wilcoxon-Mann-Whitney tests. Univariate logistic regression was used to identify independent factors associated with the use of the PAL. All significant predictors ($p < 0.20$) were then included in the final multiple logistic regression, with a stepwise backward exclusion of factors with p values of >0.05. Statistical analysis was performed using Stata version 16.

Ethics approval

The PAL was included in the MIS BAIR ethical and governance approval from Mercy Health Human Research Ethics Committee (HREC, No. R12-28) and Royal Children's Hospital HREC (No. 33025) with additional governance approval from Barwon Health and St John of God, Geelong, Victoria. Signed informed consent was obtained from the parents of all participants.

Patient and Public Involvement Statement

The MIS BAIR PAL was developed with the interests of the public and the MIS BAIR participants at the forefront of the study design. The primary outcome was to develop a PAL and observe parental use with the aim of improving patient and parental engagement. Patients were not directly involved in the development of the study design and future analyses aim to further investigate parental experience and satisfaction with the PAL.

Results

A total of 1246 participants were included in the final analysis and 26 were lost to follow-up after randomisation (2.0%) (Figure 1). The demographics of the study population are detailed in Table 1. There was an approximately even distribution of sex of the infant and their BCG vaccination status. Most participants were born via vaginal delivery, and most were breastfed.

Confidential: For Review Only

A total of 586 calls were recorded during the study. A total of 230 parents (18.5%) called the PAL at least once during the first 2 years of their participation in the trial. Of these, 69 (30%) contacted the PAL on only one occasion (inclusive of subsequent follow-up calls) and 161 (70%) on multiple occasions.

Reasons for call

Figures 2 and 3 illustrate the age distribution and the principal concerns for the PAL calls. The most common reason for a call was a rash or skin condition (87 calls, 24.5%), with calls distributed equally across infancy. The second most common reason was for a concern relating to feeding, sleeping or crying (49 calls, 14.6%), with most occurring before 20 weeks of age. A total of 34 (10.1%) and 30 calls (9.0%) were related to changes in bowel habit or vomiting, respectively, with most calls received during the neonatal period. Calls related to respiratory symptoms (26 calls, 7.8%) had a bi-modal presentation, clustered around 10 and 60 weeks of age. There were 8 calls (2.4%) relating to concerns about lumps/bumps/masses, all received in the first year of life. A total of 12 calls (3.6%) were for fever. Of the 87 dermatological-related calls, 3 (0.9%) were associated with a fever. Of the 26 respiratory symptom-related calls, 4 (1.2%) were associated with a fever. Developmental or neurological concerns (21 calls, 6.3%) occurred in early infancy, as did growth concerns (4 calls, 1.2%). Calls listed as “Other medical concern – baby unwell” included 32 calls (9.6%) for various concerns including ear/nose/throat issues (12 calls, 3.6%), abnormal pathology results or medication queries. A total of 37 calls (11.0%) were for general advice, mainly related to travel (9 calls, 2.7%), obtaining vaccines (12 calls, 3.6%), and introduction of solids to the infant’s diet (12 calls, 3.6%).

Seasonal variability

Reasons for PAL calls showed seasonal variability (Figure 4). Calls occurred most frequently during winter (31.5% of all calls), followed by autumn (24.5%), spring (22.3%), and least frequently during summer (21.7%). There was a larger number of calls for rashes and other skin conditions during winter (31.7% of all calls regarding a rash) and summer (29.3%), compared to during autumn (20.7%) and spring (18.3%). Calls related to feeding, sleeping, crying, changes of

Confidential: For Review Only

bowel habit, vomiting or respiratory symptoms were more numerous during autumn and winter. Travel advice was sought most commonly during winter. However, variability between seasons for all reasons for PAL calls were statistically insignificant on chi-square testing of proportions. Participant recruitment was approximately evenly distributed across all seasons, accounted for as a potential confounding factor, shown in Appendix A.

Call outcomes

Of the 586 calls to the PAL, 314 (53.6%) of the parents' concern(s) were resolved in a single call, 185 (31.7%) in 2 calls, and 87 (14.8%) in 3 or more calls. For the 268 calls for which this information was available, the outcomes were verbal medical advice given by a paediatrician (141 calls, 52.6%), verbal advice from a paediatric nurse or midwife (60 calls, 22.4%), written advice from a PAL clinician (email or text message) (18 calls, 6.7%), referral to be seen in-person by a paediatrician (11 calls, 4.1%), and referral to a general practitioner (31 calls, 11.6%) or the emergency department (7 calls, 2.6%). Advice was either given during the initial call or shortly after if advice from a more senior clinician was required.

Sociodemographics of PAL callers

The sociodemographic characteristics of PAL callers and non-callers were compared (Table 1). PAL callers had a slightly higher maternal age (mean 33.3 years, SD 4.1) compared with non-callers (mean 32.6 years, SD 4.9, p=0.06, t-test). First-time parents were more likely to call the PAL than non-first-time parents (140/649 (21.6%) vs. 90/597 (15.1%), p=0.003, chi^2 test). Parents of infants conceived though IVF or donor gametes were also more likely to call (8/20 (40%) vs. 49/333 (14.7%), p=0.003, chi^2 test), as were parents of infants who were breastfed (187/927 (20.2%) vs. 2/37 (5.4%), p=0.03, Fisher's exact test) and whose mothers were university-educated (185/848 (22%) vs. 44/395 (11%), p<0.001, chi^2 test). There were no statistically significant differences between the PAL caller and non-caller groups in relation to sex of the infant, mode of delivery, birthweight, maternal birthplace, or BCG vaccination status.

Multivariate analysis

Confidential: For Review Only

Univariate and multiple logistic regression analyses are outlined in Table 2. In the multiple logistic regression analysis, being a first-time parent remained a statistically significant predictor for calling the PAL (OR 1.4, 95% CI 1.1 – 1.9), as did maternal attainment of a university degree (OR 3.3, 95% CI 1.3 – 8.4). Breastfeeding and higher maternal age were not retained in the final multivariate analysis as they were no longer predicting the outcome, likely due to them being confounded by higher maternal education status in the model.

Discussion

Paediatric advice services provide guidance for parents, enhance clinician-patient communication, and improve parental satisfaction and accessibility to non-urgent health advice. (2-4) Given approximately 1 in 5 parents used the PAL, with a high proportion of parents calling multiple times, this highlights a demand for non-urgent medical advice during early childhood. Whilst studies have shown a growing demand for medical triage and assessment hotlines, this has not been studied in a clinical trial setting. (5)

The PAL operated via a single mobile phone attended by a clinical staff member during standard business hours on a voluntary rotating roster basis. The overall cost was minimal, with one-off costs for a mobile device and telephone number, given the voluntary basis of the team and the operation during business hours. Given 46.4% of PAL calls required a follow-up consultation, continuity of care was one challenge faced by PAL clinicians. However, detailed clinical documentation with all follow-up consultations occurring within one business day allowed for a streamlined process. Telephone consultation without real-time visualisation of a dermatological complaint provided some difficulty in clinical assessment. Photographs provided by parents assisted in clinical decision-making. However, using a live audio-visual telehealth service in future studies may ameliorate this challenge.

Confidential: For Review Only

In our study, the majority of concerns could be addressed by PAL staff alone and the large majority of concerns were resolved within 2 telephone calls. This is likely to have reduced the demand on emergency services for non-urgent concerns. Factors including the parent's perception of urgency, uncertainty in their own assessment of the child's symptoms and unawareness of service availability all influence a parent's decision to seek help. (6) Providing a PAL for non-urgent concerns provides an accessible means for parents to understand the importance of their child's symptoms and better inform parental decision-making in relation to whether they need to seek further help.

Dermatological concerns were the most common reason to contact the PAL across all ages, with a proclivity for summer and winter months. Many dermatological conditions, such as miliaria, atopic dermatitis and viral exanthems, present frequently during early childhood, often with seasonal variability. (7-9) Calls for respiratory concerns, gastrointestinal symptoms and disturbances with sleeping, feeding and crying were all clustered within the first 20 weeks of life; this is most likely due to early parenthood anxiety, given the high prevalence of first-time and breastfeeding parents amongst the PAL callers. The increased frequency of these calls during the winter months most likely correlates with higher incidence rates of bronchiolitis, croup and viral infections, with associated gastrointestinal upset and irritability. (10, 11) The clustering of "other" medical concerns in well infants around 30 weeks of age may correspond with the introduction of solids into the diet, often a time of apprehension. (12) Only a small proportion of calls were related to febrile episodes, even accounting for fever listed as an associated symptom rather than the main presenting concern. This may be due to parents presenting to a medical setting directly, recognising it as a potential emergency. (13)

PAL callers were more likely to be older mothers, first-time parents, university-educated and/or have used IVF or donor gametes compared to non-callers. This is consistent with literature indicating that first-time mothers of a higher maternal age have increased levels of psychological stress and anxiety during both pregnancy and the post-partum period. (14)

Confidential: For Review Only

Pregnancies acquired with the use of IVF and oocyte/sperm donation are more likely to develop stress and anxiety, (17) partly as a result of the greater risk of antenatal and intra-partum complications. (15, 16) Mothers with a university education might also feel more empowered to ask for advice than those without or those with a language barrier. Further investigation of the influence of other socioeconomic factors, such as health literacy, income and employment status, on the willingness to access and use the advice line is required as this study suggests lower educational attainment may be a barrier to PAL use.

Whilst patient satisfaction is associated with the availability and use of medical advice lines, (4) a limitation of our study is the lack of data on the influence of the PAL availability on parents' willingness to take part in the MIS BAIR trial, their satisfaction and retention rates. Future studies should address whether advice lines provide an effective incentive to improve trial participation and/or retention, and seek parents' feedback and evaluation of their benefit. Questionnaires and cluster randomised cohort studies may be of use. Additionally, our results may not be generalisable as the parents, having self-selected to participate in a trial, may not be representative of the wider population. Future PAL services could be improved by having a pre-planned referral pathway and information brochure available for common parental concerns.

Paediatric advice services are well-recognised as valuable and convenient means of empowering parents and reducing parental concerns, helping to inform decision-making and parental help-seeking. (4) The MIS BAIR PAL study demonstrates that demand for such a service exists within a clinical trial setting and is likely to be used by parents during early parenthood. Additionally, this prospective cohort study, embedded within a host RCT, adds to the evidence base for trialists conducting a Study Within A Trial (SWAT), informing design and conduct of future trials. (18)

Funding:

Confidential: For Review Only

The MIS BAIR trial is funded by research grants from the National Health and Medical Research Council of Australia (GNT1051228 and GNT1099676), The University of Melbourne, The Royal Children's Hospital Foundation, the Murdoch Children's Research Institute, Mercy Health, the Myers Foundation, the European Society of Pediatric Infectious Diseases, the Nossal Institute for Global Health, and the Aranday Foundation. Dr Laure Pittet is supported by the Swiss National Science Foundation (Early Postdoc Mobility Grant number P2GEP3_178155).

Contribution Statement:

JC and JK contributed equally to this paper and are joint first authors. JC and JK transcribed the call data collected, performed statistical analysis and wrote the manuscript. NC and LP contributed equally to this paper and are joint last authors. NC and LP were integral to the study funding, design and manuscript production. All other authors were integral in data collection and management of the paediatric advice line, including taking calls and providing advice where necessary.

Acknowledgments:

The authors thank the MIS BAIR participants and their families for their involvement in the trial. We also thank our recruitment and follow-up teams.

Conflict of Interest:

There are no reportable conflicts of interest.

References:

Confidential: For Review Only

1. Messina NL, Gardiner K, Donath S, Flanagan K, Ponsonby AL, Shann F et al. Study protocol for the Melbourne Infant Study: BCG for Allergy and Infection Reduction (MIS BAIR), a randomised controlled trial to determine the non-specific effects of neonatal BCG vaccination in a low-mortality setting. BMJ open. 2019 Dec 1;9(12):e032844.
2. Wang CJ, Ma J, Zuckerman B, Car J. The Opportunities for Telehealth in Pediatric Practice and Public Health. Pediatr Clin North Am. 2020 Aug;67(4):603-611. doi: 10.1016/j.pcl.2020.03.001.
3. Siddiqui, N., D. Greenfield and A. Lawler. Calling for confirmation, reassurance, and direction: Investigating patient compliance after accessing a telephone triage advice service. Int. J. Health Plan. Manag. 2020 May;35(3): 735-745. DOI: https://dx.doi.org/10.1002/hpm.2934
4. Li L, Georgiou A, Xiong J, Byrne M, Robinson M, Westbrook JI. Healthdirect's After Hours GP Helpline-A Survey of Patient Satisfaction with the Service and Compliance with Advice. InHIC 2016 Jan 1 (pp. 87-92).
5. Gibson A, Randall D, Tran DT, Byrne M, Lawler A, Havard A et al. Emergency Department Attendance after Telephone Triage: A Population-Based Data Linkage Study. Health services research. 2018 Apr;53(2):1137-62.
6. McLauchlan K, Ramlakhan S, Irving A. Why do parents present to the Paediatric Emergency Department with conditions suitable for management in less acute settings? A qualitative study. Eur J Emerg Med. 2020 Feb 1;27(1):40-5.
7. Kutlubay Z, Tanakol A, Engyn B, Onel C, Symsek E, Serdaroglu S et al. Newborn skin: common skin problems. Maedica. 2017 Jan;12(1):42.
8. Foley P, Zuo Y, Plunkett A, Marks R. The frequency of common skin conditions in preschool-age children in Australia: atopic dermatitis. Arch. Dermatol. 2001 Mar 1;137(3):293-300.
9. Foley P, Zuo Y, Plunkett A, Merlin K, Marks R. The frequency of common skin conditions in preschool-aged children in Australia: seborrheic dermatitis and pityriasis capitis (cradle cap). Arch. Dermatol. 2003 Mar 1;139(3):318-22.

Confidential: For Review Only

10. Lipsett SC, Monuteaux MC, Fine AM. Seasonality of common pediatric infectious diseases. Pediatr Emerg Care. 2021 Feb 1;37(2):82-5.

11. Hoeppner T, Borland M, Babl FE, Neutze J, Phillips N, Krieser D et al. Influence of weather on incidence of bronchiolitis in Australia and New Zealand. J Paediatr Child. 2017 Oct;53(10):1000-6.

12. Arora A, Manohar N, Hector D, Bhole S, Hayen A, Eastwood J et al. Determinants for early introduction of complementary foods in Australian infants: findings from the HSHK birth cohort study. Nutrition journal. 2020 Dec;19(1):1-0.

13. Bereznicki BJ, Tucker MG, Beggs SA, Zosky GR, Bereznicki LR. Emergency department presentations of febrile children to an Australian public hospital. J Paediatr Child. 2018 Dec;54(12):1308-13.

14. Aasheim V, Waldenström U, Hjelmstedt A, Rasmussen S, Pettersson H, Schytt E. Associations between advanced maternal age and psychological distress in primiparous women, from early pregnancy to 18 months postpartum. BJOG BJOG-INT J OBSTET GY. 2012 Aug;119(9):1108-16.

15. Berntsen S, Larsen EC, la Cour Freiesleben N, Pinborg A. Pregnancy outcomes following oocyte donation. Best Pract. 2021 Jan 1;70:81-91.

16. Wertheimer A, Melamed S, Ashwal E, Sapir O, Oron G, Ben-Haroush A et al. Complications of the third stage of labor are more prevalent in IVF pregnancies. J. Matern. 2020 Feb 21:1-5.

17. Hammarberg K, Fisher JR, Wynter KH. Psychological and social aspects of pregnancy, childbirth and early parenting after assisted conception: a systematic review. Hum. Reprod. 2008 Sep 1;14(5):395-414.

18. Treweek S, Bevan S, Bower P, Campbell M, Christie J, Clarke M, Collett C, Cotton S, Devane D, El Feky A, Flemyng E. Trial forge guidance 1: what is a study within a trial (SWAT)?. Trials. 2018 Dec;19(1):1-5.

Confidential: For Review Only

Confidential: For Review Only

Table 1. PAL study parent and infant demographics.

	All participants (n=1246)	PAL callers (n=230)	PAL non-callers (n=1016)	P-value
**Sex of child**				
**Male**	628 (50.4%)	117 (50.9%)	511 (50.3%)	0.9
**Female**	618 (49.6%)	113 (49.1%)	505 (49.7%)	
**First time parent**				
**Yes**	649 (52.1%)	140 (60.9%)	509 (50.1%)	0.003
**No**	597 (47.9%)	90 (39.1%)	507 (49.9%	
**Number of siblings**				
**0**	649 (52.1%)	140 (60.9%)	509 (50.1%)	0.02
**1**	388 (31.1%)	69 (30.0%)	319 (31.4%)	
**2**	150 (12.0%)	15 (6.5%)	135 (13.3%)	
**3**	34 (2.7%)	4 (1.7%)	30 (3.0%)	
**4**	13 (1.0%)	1 (0.4%)	12 (1.2%)	
**5**	10 (0.8%)	1 (0.4%)	9 (0.9%)	
**6**	0 (0.0%)	0 (0.0%)	0 (0.0%)	
**7**	2 (0.2%)	0 (0.0%)	2 (0.2%)	
**Birth method**				
**Vaginal**	800 (64.2%)	150 (65.2%)	650 (64.0%)	0.7
**Caesarean**	446 (35.8%)	80 (34.8%)	366 (36.0%)	
**Gestational age**				
**Weeks***	39.3 (38.4, 40.3)	39.4 (38.5, 40.5)	39.3 (38.4, 40.3)	0.1
**IVF or sperm/egg donor**				
**IVF/donor used**	20 (1.6%)	8 (3.5%)	12 (1.2%)	0.003
**No IVF/donor used**	333 (26.7%)	49 (21.3%)	284 (28.0%)	
**Unknown**	893 (71.7%)	173 (75.2%)	720 (70.9%)	
**Birthweight**				
**Birthweight (kg)***	3.4 (3.1, 3.7)	3.4 (3.1, 3.8)	3.4 (3.1, 3.7)	0.3
**Maternal age at delivery**				
**Years#**	32.7 (4.7)	33.3 (4.1)	32.6 (4.9)	0.06
**Mother's birthplace**				
**Australia**	902 (72.4%)	169 (73.5%)	733 (72.2%)	0.2
**Outside Australia**	344 (27.6%)	61 (26.5%)	283 (27.9%)	

Confidential: For Review Only

**Maternal education**				
**No education/up to Year 10**	69 (5.5%)	5 (2.2%)	64 (6.3%)	
**Year 12/trade**	326 (26.2%)	39 (17.0%)	287 (28.1%)	<0.001
**University**	848 (68.1%)	185 (80.4%)	663 (65.3%)	
**Unknown**	3 (0.2%)	1 (0.4%)	2 (0.2%)	
**Breastfed**				
**Breastfed**	927 (74.4%)	187 (81.3%)	740 (72.8%)	
**Not breastfed**	37 (3.0%)	2 (0.9%)	35 (3.4%)	0.03
**Unknown**	282 (22.6%)	41 (17.8%)	241 (23.7%)	
**Received BCG**				
**Yes**	622 (49.9%)	119 (51.7%)	503 (49.5%)	0.4
**No**	624 (50.1%)	111 (48.3%)	513 (50.5%)	

Reported as number (%) and p-value calculated using chi-square or Fisher's exact test, unless otherwise noted.

* Reported as median (IQR). P-value calculated using Wilcoxon-Mann-Whitney test.

[#] Reported as mean (SD). P-value calculated using independent samples t-test.

Confidential: For Review Only

Table 2. Univariate and multiple logistic regression analyses predicting PAL callers.

	Univariate model OR* (95% CI)	P-value	Multivariate model OR* (95% CI)	P-value
**Sex**				
**Male**	1.0 (0.8 – 1.4)	0.9		
**First time parent**				
**Yes**	1.5 (1.2 – 2.1)	0.003	1.4 (1.1 – 1.9)	0.02
**Number of siblings**				
**0**	Reference	Reference		
**1**	0.8 (0.6 – 1.1)	0.1		
**2**	0.4 (0.2 – 0.7)	0.002		
**3**	0.5 (0.2 – 1.4)	0.2		
**4**	0.3 (0.04 – 2.4)	0.3		
**5**	0.4 (0.05 – 3.2)	0.4		
**Birth method**				
**Vaginal**	1.1 (0.8 – 1.4)	0.7		
**Gestational age**				
**Weeks**	1.1 (1.0 – 1.2)	0.08		
**IVF or sperm/egg donor**				
**Yes**	3.9 (1.5 – 9.9)	0.005#		
**Birthweight**				
**<2500 g**	0.8 (0.3 – 1.8)	0.5		
**Birthweight (kg)**	1.2 (0.9 – 1.6)	0.2		
**Maternal age at delivery**				
**Years**	1.0 (1.0 – 1.1)	0.06		
**Mother's birthplace**				
**Australia**	1.1 (0.8 – 1.5)	0.7		
**Maternal education**				
**No education/up to Year 10**	Reference	Reference	Reference	
**Year 12/trade**	1.7 (0.7 – 4.6)	0.3	1.7 (0.6 – 4.5)	0.3
**University**	3.6 (1.4 – 9.0)	0.007	3.3 (1.3 – 8.4)	0.01
**Breastfed**				
**Yes**	4.4 (1.1 – 18.6)	0.04		
**Received BCG**				
**Yes**	1.1 (0.8 – 1.5)	0.5		

* Odds ratio calculated using logistic regression method.

Confidential: For Review Only

[#] IVF was not included in the multivariate analysis due to missing data resulting in loss of statistical power.

Confidential: For Review Only

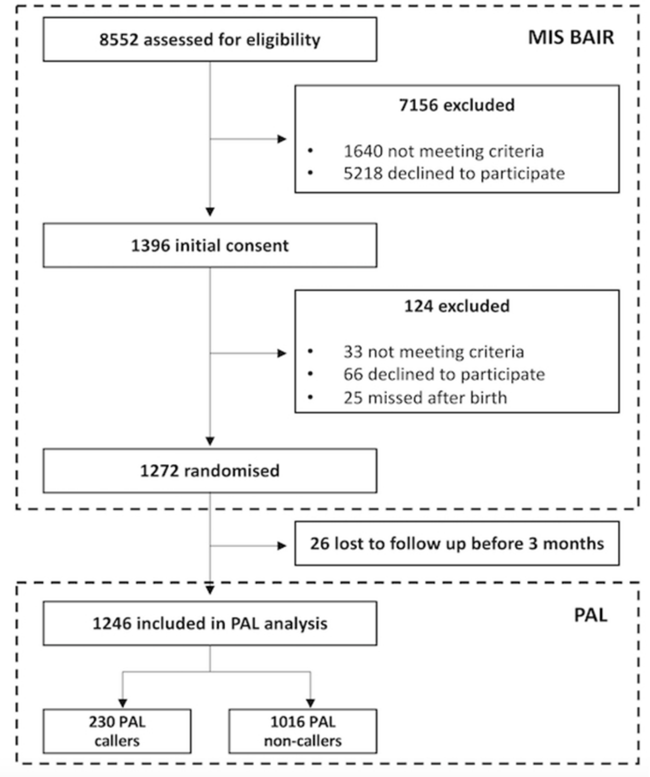

481x855mm (38 x 38 DPI)

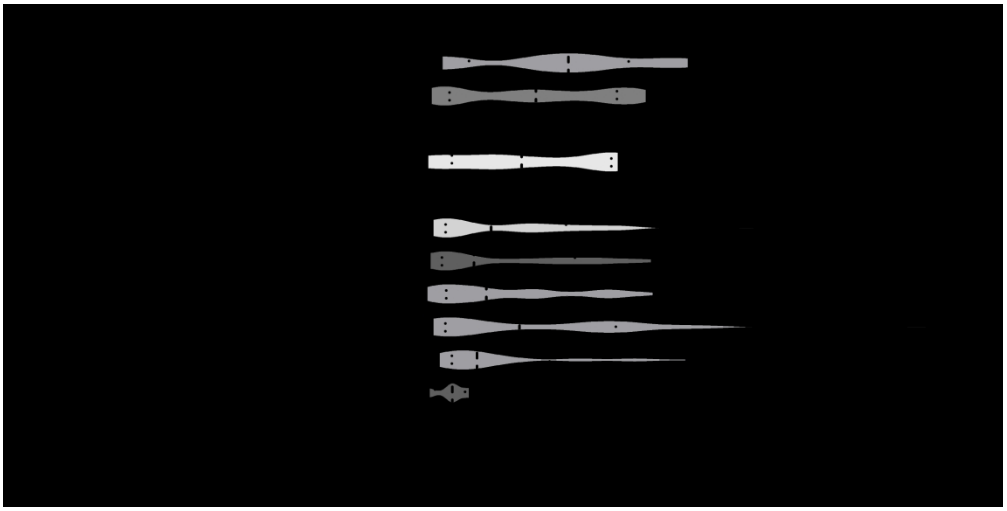

Figure 2. Age distribution of infants, in months, at the time of the parents call to the PAL categorised by reasons.

496x250mm (118 x 118 DPI)

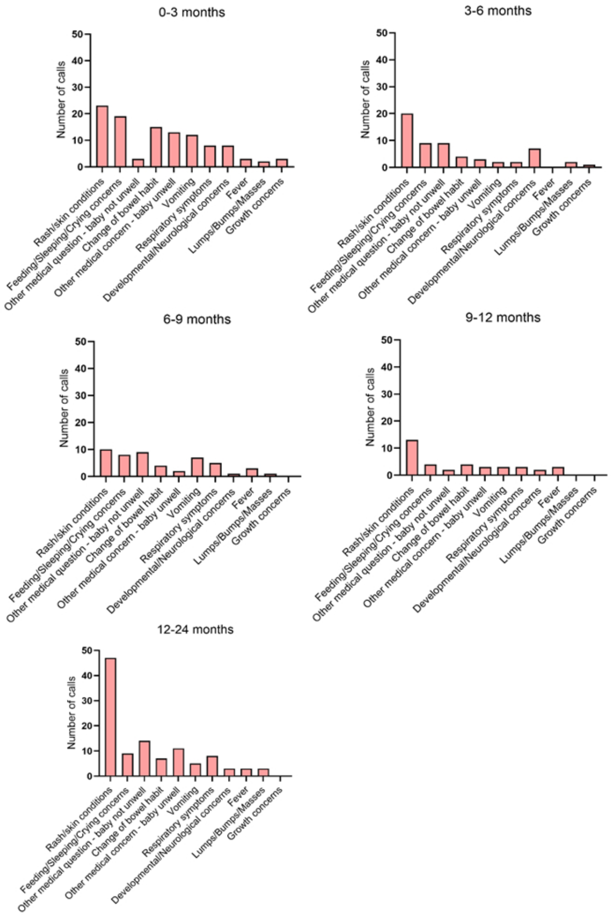

Figure 3. Frequency of PAL calls for each reason at different infant ages.

481x855mm (38 x 38 DPI)

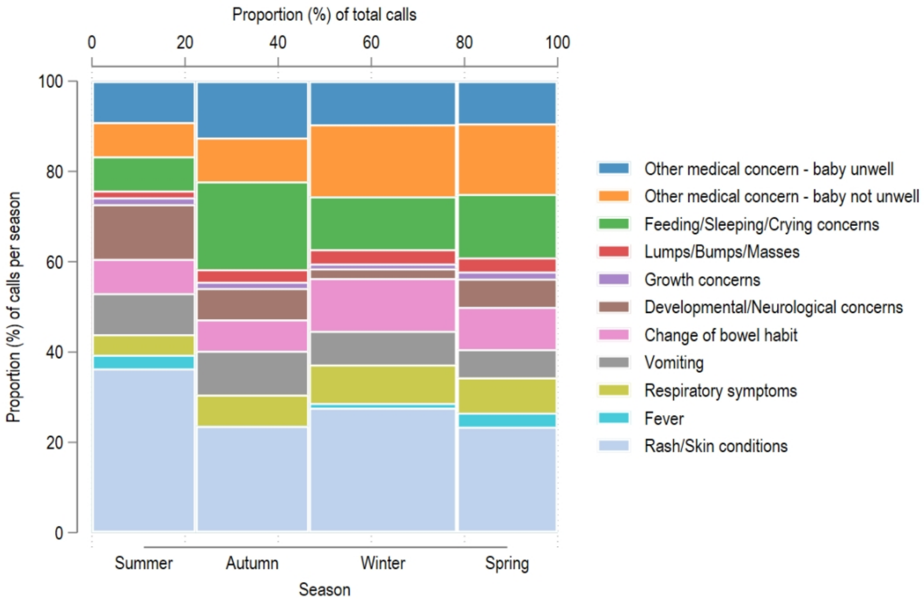

Figure 4. Seasonal distribution of calls to the PAL for each reason during the first 2 years of life. Season and the proportion of calls for each season are displayed on the x-axis. The proportion of calls for each reason within a specific season is displayed along the y-axis.

451x301mm (72 x 72 DPI)

Appendix A. Participant recruitment timing based on date of birth in 3-monthly periods.

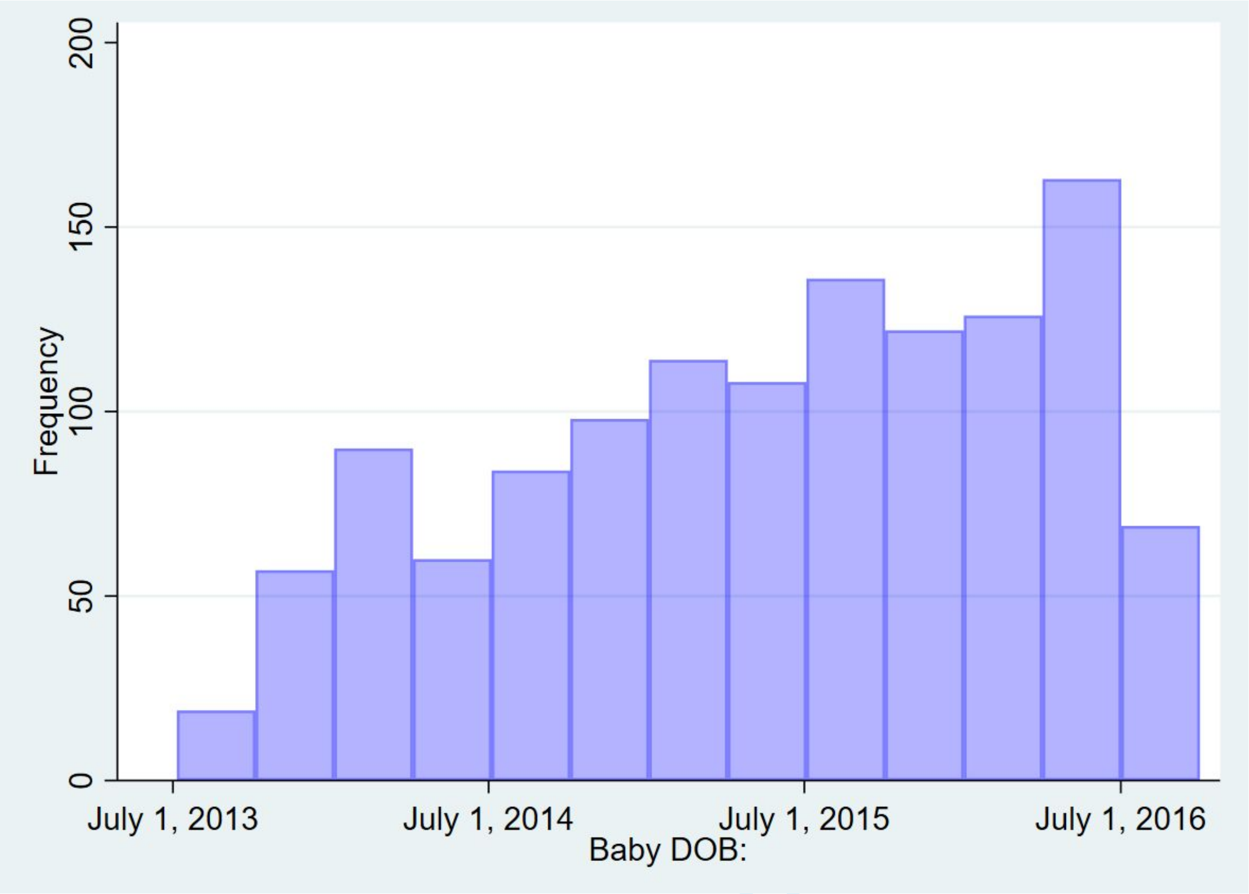

Supplement: Author's manuscript [file bmjpo-2022-001665.draft_revisions.pdf]
